# The role of response domain and scale label in the quantitative interpretation of patient-reported outcome measure response options

**DOI:** 10.1007/s11136-021-02801-9

**Published:** 2021-03-04

**Authors:** Tessa Peasgood, Jen-Yu Chang, Robina Mir, Clara Mukuria, Philip A. Powell

**Affiliations:** 1 School of Health and Related Research (ScHARR), University of Sheffield, Regent Court, 30 Regent Street, Sheffield, S1 4DA UK; 2 School of Population and Global Health, University of Melbourne, Parkville, Australia; 3 NIHR Research Design Service Yorkshire and Humber, University of Sheffield, Regent Court, 30 Regent Street, Sheffield, S1 4DA UK

**Keywords:** Patient reported outcomes (PROs), Health-related quality of life, Response options, Scale development, Scale label, Questionnaires

## Abstract

**Purpose:**

Uncertainties exist in how respondents interpret response options in patient-reported outcome measures (PROMs), particularly across different domains and for different scale labels. The current study assessed how respondents quantitatively interpret common response options.

**Methods:**

Members of the general public were recruited to this study via an online panel, stratified by age, gender, and having English as a first language. Participants completed background questions and were randomised to answer questions on one of three domains (i.e. loneliness (negatively phrased), happiness or activities (positively phrased)). Participants were asked to provide quantitative interpretations of response options (e.g. how many times per week is equal to “often”) and to order several common response options (e.g. occasionally, sometimes) on a 0–100 slider scale. Chi-squared tests and regression analyses were used to assess whether response options were interpreted consistently across domains and respondent characteristics.

**Results:**

Data from 1377 participants were analysed. There was general consistency in quantifying the number of times over the last 7 days to which each response option referred. Response options were consistently assigned a lower value in the loneliness than happiness and activities domains. Individual differences, such as age and English as a second language, explained some significant variation in responses, but less than domain.

**Conclusion:**

Members of the public quantify common response options in a similar way, but their quantification is not equivalent across domains or every type of respondent. Recommendations for the use of certain scale labels over others in PROM development are provided.

**Supplementary Information:**

The online version of this article (10.1007/s11136-021-02801-9) contains supplementary material, which is available to authorized users.

## Introduction

Patient-reported outcome measures (PROMs) are completed by patients (or proxies) in order to provide a summary of patients’ evaluation of their health or health-related quality of life. PROMs are used to assess the impact of conditions and/or interventions in the context of effectiveness studies, cost-effectiveness analysis, or to track changes in individual health in routine care [1, 2]. Evidence on their validity and reliability, for example as a function of mode of administration [3], relationship with other outcomes [4], and linguistic content [5, 6], is therefore of interest to those working in health care decision making.

PROMs consist of questions covering different domains (e.g. pain, mental health or wellbeing, physical, social, role functioning, etc.). Patients report their level of symptoms or functioning using numeric rating scales (NRS), visual analogue scales (VAS), or verbal rating scales (VRS) [7, 8]. Response options can be based on how frequently patients experience a symptom or have problems with functioning, how severe the symptom is, or on levels of difficulty (e.g. with functioning) [9]. Finally, PROM questions can also be phrased as agreement scales, for example, from strongly agree to strongly disagree [10].

The type of response option used is related to the concept being measured. For example, experience of symptoms is usually linked to either frequency or severity scales. Summary scores or weighted index values can be generated from the PROM, which can be used to assess health [11, 12]. However, verbal response options are considered to be vague quantifiers as they rely on respondents’ interpretation of terms such as ‘sometimes’ or ‘occasionally’ [13, 14]. The vagueness of verbal quantifiers is potentially problematic in the assessment of health-related quality of life, which often relies on them heavily.

Vague quantifiers are also problematic when PROMs are used by health economists to elicit health state utility values, which play an important practical role in cost-effectiveness analysis to determine health care resource allocation [15]. As response levels are displayed independently of the other response options in utility elicitation, it is important for PROMs to have response choices that are clear and can be consistently interpreted over time, context, and between people [16]. Within each type of response options, there are variations in the number of options and the qualitative labels used to distinguish between them. There are ongoing methodological uncertainties around potential differences in the interpretation of response options [13] and concerns as to whether respondents can clearly distinguish between different numbers of response categories [17, 18].

For frequency response options, the relationship between participants’ numerical estimates and corresponding linguistic terms (e.g. ‘often’, ‘some of the time’, ‘seldom’) has been explored to understand the order and the degree of difference between displayed options [13, 19–22]. While similar analysis has also been conducted on severity response options (e.g. ‘very much’, ‘quite a bit’, ‘some’) [23–25], little has been investigated regarding response options that quantify difficulties (e.g. ‘a little difficulty’, ‘moderate difficulty’) [26].

How participants assign a quantitative value to qualitative response options (e.g. how many times an event has to have happened to match the label ‘often’ or ‘sometimes’) is not clearly understood. Additionally, evidence on whether such interpretation varies across different context or domains remains scarce. The interpretation of response options could also potentially be heterogeneous within different subpopulations, for example, with regard to their health, language, and cultural background. Whether a uniformed questionnaire can provide a generally consistent measure across different groups requires exploration [27, 28].

This project aimed to explore how respondents quantitatively interpret common PROM response options. This was in part to support ongoing instrument development work of a new preference-based measure of health and wellbeing (the EQ-HWB; https://scharr.dept.shef.ac.uk/e-qaly/) and the choice of questions and response options investigated in this study was linked to those being considered for inclusion within this broader project. Three key questions were addressed: 1) whether the quantification of different response options reflects their intuitive or linguistic ordering; 2) whether response options to questions assessing different domains are interpreted differently; and 3) whether individual characteristics, such as age and having English as a second language (ESL), influence the way response options are interpreted.

## Methods

### Sample

Adult residents in the UK were recruited via the Prolific online panel [29] in January 2019 (*n* = 1401), pre-screened to cover a spread of age (18–47 versus 48 + year olds), gender, and ESL. No formal a priori sample size estimation was undertaken. Participants received £1.20 for completing the online survey.

### Survey

**Fig. 1:**
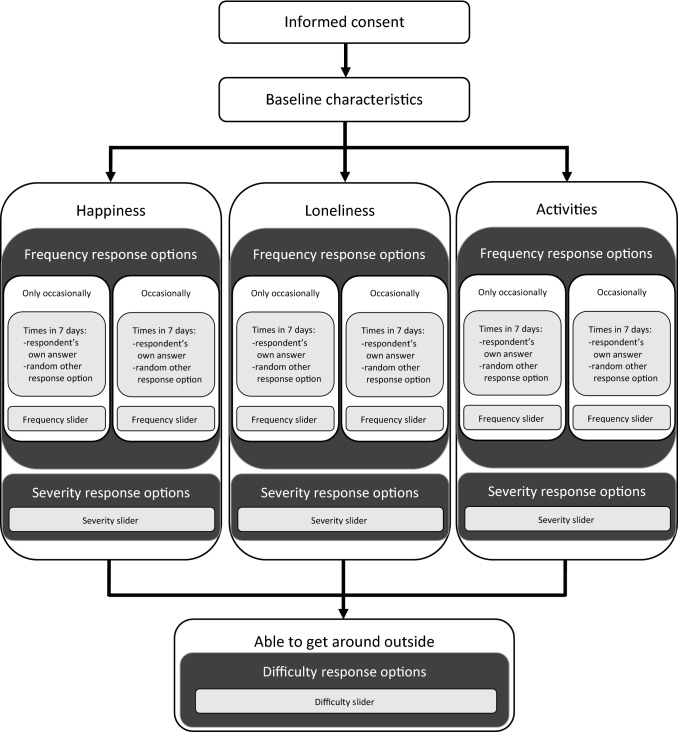
Schematic of survey flow. Respondents were randomised to answer questions on one of three health related quality of life domains (happiness, loneliness, or activities), and then to either the ‘only occasionally’ or ‘occasionally’ frequency response option. Frequency slider response options included: (‘none of the time’), ‘only occasionally’/’occasionally’, ‘sometimes’, ‘often’, ‘most of the time’, (‘all of the time’). Severity slider response options included: (‘not at all’), ‘a little bit’, ‘some’, ‘somewhat’, ‘quite a bit’, ‘very much’. Difficulty slider response options included: (‘no difficulty’), ‘slight difficulty’, ‘some difficulty’, ‘a lot of difficulty’, (‘unable’)

Ethics approval was granted from the host institution ethics committee. Figure 1 illustrates the survey flow. Following consent, participants provided background characteristics, including their age, gender, ethnic group, highest educational qualification, health, and any chronic mental or physical health problems (see the full survey in Online Resource 1). Participants were randomised to one of three domains (loneliness, happiness, or activities) and asked a series of questions about the quantitative interpretation of commonly used PROM response options. Each participant thus received a question stem that corresponded to the abovementioned domains, either: (1) a negatively phrased question related to social functioning, ‘I felt lonely’; (2) a positive phrased mental health/wellbeing question, ‘I felt happy’; or (3) an activity/role functioning question, ‘I was able to do the things I wanted to do’.

Following stratification by one of the three questions, participants were further randomised such that half were given the response option ‘occasionally’ to interpret and half were given ‘only occasionally’. Given that ‘occasionally’ and ‘only occasionally’ always fell in the same position within response options this randomisation enabled testing of whether the actual wording of the response option made a difference beyond their ranked order. ‘Occasionally’ and ‘only occasionally’ were tested in separate arms in part to inform selection of response options for the new measure.

First, participants were asked to respond to the question ‘Thinking about how things have been over the last 7 days…’ with the stem dependent on question randomisation (e.g. ‘I felt lonely’), using a 5-point scale (ranging from ‘none of the time’ to ‘most or all of the time’; for full response scales to all survey questions see Online Resource 1). Participants were then asked to provide a quantitative interpretation of their own response to this question, based on the number of days over a 7-day period they thought the response best referred to, on a 8-point scale (ranging from ‘not even once in the last 7 days’ to ‘seven or more times in the last 7 days). The same quantification question was asked again for another random response option in addition to their own answer.

In order to observe what quantitative values participants assigned to each response option, relative to one another on the same scale, participants then completed three slider tasks. First, for each set of randomly ordered frequency response options (‘occasionally’ or ‘only occasionally’, ‘sometimes’, ‘often’, ‘most of the time’^1^), participants were asked to assign a numeric value between 0 and 100 using a slider for each response option (0 = none of the time, 100 = all of the time).

Second, they were asked to assign a set of randomly ordered severity response options (‘a little bit’, ‘somewhat’, ‘some’, ‘quite a bit’, ‘very much’) on a similar 0 to 100 slider scale (0 = not at all, 100 was undefined). In both cases, these questions related to the domain to which the participant had been randomised (e.g. loneliness). For the frequency slider the top anchor ‘all of the time’ is intuitive, however, there is no such clear top anchor for a severity scale, which can be applied across the three domains. The top of the severity scale was left undefined to avoid introducing potential focusing effects from terms that are not usually part of the response option set and also to allow respondents to place ‘very much’ at the top of the scale should they wish.

Third, all participants responded to a question with a difficulty response option, linked to mobility: ‘Thinking about how things have been over the last 7 days… How well were you able to get around outside?’ on a 5-point scale (ranging from ‘no difficulty’ to ‘unable’). After this, participants were asked to assign the following response options ‘a lot of difficulty’, ‘some difficulty’ and ‘slight difficulty’ on a 0 to 100 slider scale (0 = no difficulty, 100 = unable).

To help with data quality, the survey was designed to be short (less than 10 min); respondents were timed out after 30 min. The research team included a patient researcher who was involved in supporting the design of the study, including the survey and input into the clarity and content of the study information sheet.

### Data quality

We judged a respondent to have answered in a logically inconsistent way when they gave a quantitative answer for the bottom response option (e.g. ‘only occasionally’ or ‘none of the time’) that was equal to or higher than their response to the top response option (e.g. ‘most of the time’). Inconsistent responses were dropped from within the group of questions where the inconsistency was identified (i.e. frequency quantification in number of times over 7 days, frequency slider, severity slider, or difficulty slider). However, for the selection about the number of times over 7 days, selections for the bottom and top response option that were both at the top end of the scale (7 or more times) were not considered as inconsistent due to the upper censoring of the scale. In addition to the above, we excluded completely individuals who had three or more inconsistencies in responses, as these respondents were considered to not have paid attention or understood the tasks. An additional analysis was conducted with respondents with any inconsistencies dropped to explore the effect on the results (see Online Resource 4).

### Data analysis

Descriptive analyses were conducted to document characteristics of respondents and to compare results of the slider and frequency quantification questions for different response options across different domains (e.g. happiness versus loneliness). Differences were tested using Fisher’s exact test (for medians) and unpaired t-tests (for means). We also explored descriptively the relative gaps between mean responses across the response options (no statistical test performed) and the variability of respondents’ answers for each slider response (using a variance comparison test) to indicate the consistency of interpretation of the options across our sample.

We used regression analysis to explore the combined impact of respondent characteristics and the domain of the question on the assigned values. We ran separate ordinary least squares (OLS) regressions which combined all slider answers relating to a particular response option (e.g. ‘sometimes’); this meant combining respondents from different arms of the study (for frequency and severity response options, see Fig. 1). For each model, the response option was used as the dependent variable with domain and respondent characteristics as the independent variables. We also ran the frequency and severity models without the domain variables to explore the extent of variance explained by individual characteristics. As each respondent was included only once in each model, no adjustment for clustering standard errors was used. Ideally, respondent characteristics should have no impact on interpretation of the labels; therefore, small coefficients and low variance explained by the model were preferred. Accordingly, we did not have any a priori effect sizes against which to judge the effects.

## Results

### Inconsistency checks

Of 1401 survey completions, 229 had one inconsistency, 41 had two and 24 had three or more. The latter were dropped leaving a valid sample of 1377 participants. Most remaining inconsistencies occurred in interpreting the ‘difficulty’ response options linked to the mobility question (15.6% of the remaining sample had an inconsistency on this question). For those randomised to the happiness and activities domains, the slider for the difficulty question had reverse anchors, such that to the right was the most negative (i.e. greater problems with mobility), this contrasts with earlier questions where the right anchor was the most positive (e.g. more happiness). Accordingly, inconsistencies on the difficulty question were more pronounced in the happiness (17.5% of this sub-sample) and activities (22.9% of this sub-sample) groups, than the loneliness group (6.5% of this sub-sample).

### Respondent characteristics

Basic characteristics of the valid sample are in Table 1. Of the 1377 included completions, 53.1% were female, 37.5% reported a health condition, and 85.6% reported English as their first language. The mean age of the sample was 42.4 years (SD = 14.0), with a minimum of 18 and maximum of 86 years. On average, participants completed the survey in 6.9 min (SD = 3.4), with a minimum of 2.2 and a maximum of 30.2 min. Respondents were evenly randomised into three groups (i.e. happiness *n* = 458, loneliness *n* = 460, and activity *n* = 459), and there were no significant differences in characteristics between groups (see Table 1).

**Table 1:** Respondents’ characteristics

Characteristic (N (%), unless otherwise specified)	Total study population^b^ (*N* = 1377)	Domains			*p* value of test of difference between subgroups^a^
		Happiness (*N* = 458)	Loneliness (*N* = 460)	Activities (*N* = 459)	
Age* (Mean (SD))	42.4 (14.0)	42.2 (13.5)	42.9 (14.4)	42.2 (14.0)	.700
Duration of survey (minutes) (Mean (SD))	6.9 (3.4)	6.8 (3.3)	6.9 (3.3)	7.1 (3.6)	.317
Gender					.655
Female	731 (53.1)	245 (53.5)	241 (52.4)	245 (53.4)	
Male	642 (46.6)	211 (46.1)	218 (47.4)	213 (46.4)	
Other	3 (0.2)	2 (0.4)	1 (0.2)	0 (0.0)	
Prefer not to say	1 (0.1)	0 (0.0)	0 (0.0)	1 (0.1)	
Has caring responsibilities	182 (13.2)	60 (13.1)	60 (13.0)	63 (13.5)	.975
Has English as a second language	198 (14.4)	65 (14.2)	65 (14.1)	68 (14.8)	.948
Health status					.454
Very good	247 (17.9)	89 (19.4)	87 (18.9)	71 (15.5)	
Good	718 (52.1)	234 (51.1)	237 (51.5)	247 (53.8)	
Fair	318 (23.1)	105 (22.9)	106 (23.0)	107 (23.3)	
Bad	87 (6.30)	27 (5.9)	26 (5.7)	34 (7.4)	
Very bad	7 (0.5)	3 (0.7)	4 (0.9)	0 (0.0)	
Health interferes with activities					.923
Not at all	790 (57.4)	267 (58.3)	259 (56.3)	264 (57.5)	
A little bit	384 (27.9)	119 (26.0)	139 (30.2)	126 (27.5)	
Moderately	109 (7.9)	37 (8.1)	34 (7.4)	38 (8.3)	
Quite a bit	70 (5.1)	25 (5.5)	22 (4.8)	23 (5.0)	
Extremely	24 (1.7)	10 (2.2)	6 (1.3)	8 (1.7)	
Highest educational qualification					.335
Bachelors or equivalent first degree level qualification or higher	710 (51.6)	242 (52.8)	237 (51.5)	231 (50.3)	
A level or equivalent post-secondary level qualification	394 (28.6)	123 (26.9)	136 (29.6)	135 (29.4)	
GCSE or equivalent secondary school qualification	251 (18.2)	88 (19.2)	75 (16.3)	88 (19.2)	
None of the above	22 (1.6)	5 (1.1)	12 (2.6)	5 (1.1)	
Ethnicity					.304
Asian/Asian British	71 (5.2)	24 (5.2)	23 (5.0)	24 (5.2)	
Black/African/Caribbean/Black British	22 (1.6)	4 (0.9)	6 (1.3)	12 (2.6)	
Mixed/Multiple ethnic groups	26 (1.9)	6 (1.3)	6 (1.3)	14 (3.1)	
Other White	153 (11.1)	51 (11.1)	51 (11.1)	51 (11.1)	
Other ethnic group	10 (0.7)	4 (0.9)	2 (0.4)	4 (0.9)	
White British	1,093 (79.4)	368 (80.3)	372 (80.9)	353 (76.9)	
Prefer not to say	2 (0.1)	1 (0.2)	0 (0.0)	1 (0.2)	
Health condition present					.188
Yes	516 (37.5)	180 (39.3)	179 (38.9)	157 (34.2)	
Mental health condition	240 (17.4)	89 (19.4)	85 (18.5)	66 (14.4)	.100
Physical health condition	423 (30.7)	142 (31.0)	149 (32.4)	132 (28.8)	.484
No	851 (61.8)	274 (59.8)	276 (60.0)	301 (65.6)	
Prefer not to say	10 (0.7)	4 (0.9)	5 (1.1)	1 (0.2)	

*Note* ANOVA: analysis of variance

*One respondent recorded an age less than 18 years, which was recoded as missing (in the total population and in the subset of activity domain). Respondents were randomly assigned to one of the three different domains to answer survey questions (see Fig. 1)

^a^ANOVA or chi-square test for continuous (Prob > F) or categorical (Pr) variables, respectively.

^b^Individuals who had three or more inconsistencies in responses were excluded as these respondents were considered to not have paid attention or understood the tasks

### Response option interpretation based on number of times experienced

Table 2 shows the median response to interpretations based on the number of times over the last 7 days their own selection refers to and similarly for a randomly specified ‘other’ response option. The median response increases in line with expectations.

**Table 2:** Median number of times over the last 7 days participants reported that each possible frequency response referred, split by occasionally and only occasionally arm

Domain	Only occasionally		Occasionally		Fisher’s exact test (one tailed)
	Median	*N*	Median	*N*	*p*
Lonely					
Only occasionally/occasionally	2	108	2	112	.074
Sometimes	3	100	3	93	.838
Often	5	79	5	77	.603
Most or all of the time	6	72	6	69	.788
Activities					
Only occasionally/occasionally	2	86	3	91	.169
Sometimes	4	98	3	97	.998
Often	5	121	5	106	.478
Most or all of the time	7	148	7	157	.831
Happiness					
Only occasionally/occasionally	2	101	3	73	**.032**
Sometimes	3	123	4	96	**.003**
Often	5	129	5.5	120	.795
Most or all of the time	7	111	7	99	.491

*Note* N varies because respondents were asked to interpret their own answer to the domain-specific question and due to randomisation of the remaining other responses. Participants were randomised into either the ‘occasionally’ arm or the ‘only occasionally arm’, in addition to being randomised to ‘happiness’, ‘lonely’ and ‘activity’ arms. Test of differences in the distributions of responses between the occasionally and only occasionally arms (Fisher’s exact test), *p* values < .05 in bold

Comparisons between the ‘only occasionally’ arm and the ‘occasionally’ arm found significant differences for interpretations of the responses to the ‘felt happy’ question for ‘occasionally/only occasionally’ (*p* = 0.032 one tailed Fisher’s exact test) and ‘sometimes’ (*p* = 0.003 one tailed Fisher’s exact test). All other comparisons between these arms were not significant. This means the interpretation of ‘sometimes’ for the happy question is lower when presented alongside ‘only occasionally’ as the next response choice.

### Response option interpretation based on sliders

**Fig. 2:**
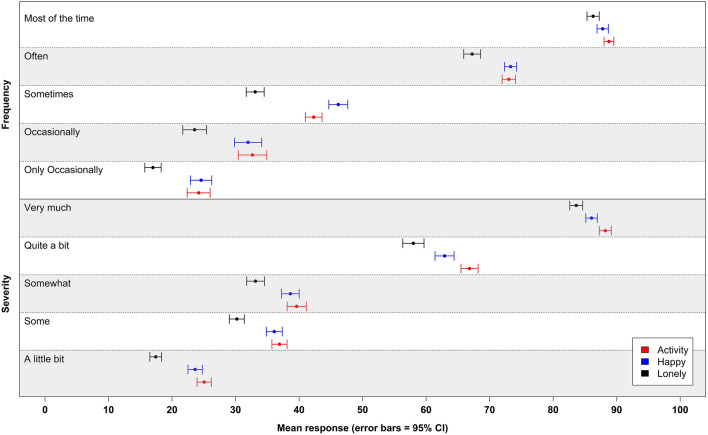
Mean value to slider questions for frequency and severity response options. Legend value attributed by participants to each response option on a slider task across ‘activities’, ‘happiness’ and ‘loneliness’ quality of life domains. Response options were presented simultaneously in slider tasks with either a frequency (‘occasionally’ or ‘only occasionally’, ‘sometimes’, ‘often’, ‘most of the time’) or severity (‘a little bit’, ‘somewhat’, ‘some’, ‘quite a bit’, ‘very much’) response option scale

Figure 2 shows the mean answer on the sliders by question, for each response option (see Online Resource 2 for a table of this data). ‘Only occasionally’ is significantly lower than ‘occasionally’ for all three questions (unpaired t-test, for lonely *t* = − 5.698 *p* < 0.001, happy *t* = − 5.364 *p* < 0.001, and activities *t* = − 5.798 *p* < 0.001). For other response options, those presented within the ‘only occasionally’ versus ‘occasionally’ arm were not significantly different from one another, with one exception (the response of ‘most of the time’ within the activities domain; unpaired t-test, *t* = -2.562, *p* = 0.005), and consequently answers to sliders are shown combined across these two arms (except for the only occasionally/occasionally option).

The graph shows a significantly lower quantitative interpretation of response options in the loneliness domain compared to the happiness and activities domains (as indicated by non-overlapping 95% confidence intervals), with the exception of ‘most of the time’, where the confidence intervals overlap between the loneliness and happiness domains.

The ordinal interpretation of response options is in line with expectations. ‘Occasionally’ was interpreted as quantitatively greater than ‘only occasionally’. There are some interesting differences between the frequency and severity terms: ‘a little bit’ is quantitatively interpreted as being closer to ‘only occasionally’ than ‘occasionally’. ‘Often’ was given a higher score than the fourth severity category, ‘quite a bit’. The response option ‘some’ was interpreted similarly to ‘somewhat’, although the SD for ‘somewhat’ was higher for all three domains (variance comparison test: happy f = 0.821, 2*Pr (F < f) = 0.039, activities f = 0.630, 2*Pr (F < f) < 0.001, lonely f = 0.703, 2*Pr (F < f) < 0.001). There is a greater distance between the third (‘often’/’quite a bit’) and the second response options (‘sometimes’/’somewhat’) than for other differences.

The slider responses to the difficulty mobility question (i.e. ‘I was able to get around outside with…’) shows that the terms ‘a lot of difficulty’ (*M* = 85.7, SD = 10.6); ‘some difficulty’ (*M* = 49.5, SD = 17.2); and ‘slight difficulty’ (*M* = 26.7, SD = 16.2) were interpreted broadly as expected.

### Response option interpretation based on regression analysis

Table 3 shows the predictive models for the slider responses to frequency and severity response options regressed on respondent characteristics across the three randomised domains (i.e. loneliness, happiness, activities). Respondents gave a statistically significant higher value to all response options in the happiness and activities domains than loneliness domains, and these effects remain after individual characteristics are controlled for.

**Table 3:** OLS regression results for respondent characteristics predicting slider responses to frequency and severity response options

Characteristics	Frequency response options					Severity response options				
	Only occasionally	Occasionally	Sometimes	Often	Most of the time	A little bit	Somewhat	Some	Quite a bit	Very much
Number of observations	667	660	1330	1325	1329	1338	1338	1,338	1338	1356
Domain										
Happiness (Ref: loneliness)	7.662*** (1.141)	8.206*** (1.488)	13.050*** (1.007)	6.043*** (0.803)	1.497* (0.629)	6.108*** (0.759)	5.577*** (1.025)	5.843*** (0.871)	5.043*** (1.071)	4.495*** (0.811)
Activities (Ref: loneliness)	7.322*** (1.156)	8.665*** (1.497)	9.022*** (1.014)	5.767*** (0.807)	2.455*** (0.633)	7.502*** (0.755)	6.597*** (1.019)	6.681*** (0.866)	9.117*** (1.065)	6.726*** (0.806)
Respondent characteristics										
Age (continuous)	− 0.052 (0.036)	− 0.107* (0.047)	− 0.046 (0.032)	0.054* (0.025)	− 0.0143 (0.0198)	− 0.121*** (0.024)	0.092** (0.032)	− 0.049 (0.027)	0.102** (0.033)	0.051* (0.025)
Female (Ref: male, other, prefer not to say)	− 0.918 (0.951)	0.120 (1.247)	2.321** (0.842)	1.655* (0.670)	1.831*** (0.525)	− 0.042 (0.630)	− 1.376 (0.850)	0.300 (0.723)	1.451 (0.888)	2.068** (0.675)
English as a second language (Ref: English native speakers)	3.306* (1.398)	1.448 (1.822)	0.069 (1.233)	− 0.592 (0.983)	− 0.852 (0.771)	0.256 (0.932)	− 3.100* (1.259)	− 1.041 (1.070)	− 6.078*** (1.315)	− 0.466 (1.000)
With mental health conditions (Ref: no mental health conditions)	− 0.149 (1.345)	0.018 (1.841)	− 1.909 (1.216)	0.260 (0.968)	− 0.542 (0.758)	0.586 (0.919)	1.380 (1.242)	− 0.133 (1.056)	2.955* (1.298)	1.088 (0.982)
With physical health conditions (Ref: no physical health conditions)	− 0.674 (1.128)	− 2.536 (1.522)	− 0.982 (1.014)	− 0.547 (0.807)	0.158 (0.632)	− 0.766 (0.757)	− 0.653 (1.022)	− 0.036 (0.869)	0.134 (1.068)	− 0.576 (0.809)
With bachelors or higher degrees (Ref: with education below degree level)	− 2.767** (0.962)	− 2.804* (1.233)	− 1.062 (0.838)	0.054 (0.668)	0.0183 (0.523)	− 1.400* (0.627)	− 0.283 (0.847)	0.633 (0.720)	− 1.707 (0.885)	0.496 (0.672)
Constant	20.87*** (1.952)	30.17*** (2.449)	35.04*** (1.690)	64.27*** (1.345)	86.11*** (1.055)	23.48*** (1.265)	30.45*** (1.708)	32.00*** (1.452)	54.00*** (1.785)	78.09*** (1.352)
Adjusted R^2^	0.090	0.081	0.123	0.054	0.0154	0.099	0.045	0.048	0.083	0.055
* F*-test (Prob > F)	9.218 (.000)	8.262 (.000)	24.350 (.000)	10.450 (.000)	3.601 (.000)	19.420 (.000)	8.910 (.000)	9.476 (.000)	16.200 (.000)	10.850 (.000)

*Note*: Standard errors in parentheses

*OLS* ordinary least squares. *Ref* reference category

***p < 0.001, **p < 0.01, *p < 0.05. Each column represents a separate regression model for a single response option and the number of observations vary between different response options primarily due to the randomisation in the survey (see Fig. 1)

Older participants tended to give more polarised responses, giving a lower value for lower anchored response options (e.g. ‘occasionally’) and a higher value for higher anchored response options (e.g. ‘often’) than younger participants. Women gave higher values for some of the response options at the top of the scale (i.e. ‘sometimes’, ‘often’, ‘most of the time’, ‘very much’). Participants with a degree gave lower values for ‘a little bit’, ‘occasionally’ and ‘only occasionally’. Those disclosing a mental health problem gave a higher value for ‘quite a bit’ than those not disclosing a problem.

Finally, participants with ESL reported a higher value for ‘only occasionally’ and lower values for ‘quite a bit’ and ‘somewhat’. Tests of equality of variance between slider values from participants with English as a first language versus ESL finds a significantly greater variance for ESL for ‘quite a bit’ across all three domains (variance comparison test: happy f = 0.422, 2*Pr(F < f) < 0.001, lonely *f* = 0.519 2*Pr(F < f) < 0.001, activities *f* = 0.511, Pr(F < f) < 0.001), and for ‘very much’ in the loneliness domain (*f* = 0.489, 2*Pr(F < f) < 0.001). No other significant differences were observed. Overall minimal variance in slider values is explained by respondent characteristics. When question type is not included as a covariate, the highest adjusted R-squared for the severity responses is 0.034 and 0.024 for the frequency responses (see Online Resource 3). Accordingly, the biggest variation in responding is driven by context (or the domain being measured).

Table 4 shows how well the respondent characteristics predict the slider response for the difficultly response options linked to the mobility question. Only a small amount of the variation in slider responses was explained by respondent characteristics (between 0.4 and 3.0%). Women provided a significantly higher slider value (i.e. closer to ‘unable’) for all levels of difficulty. Those declaring a physical health problem and those who were older, interpreted ‘slight difficulty’ as quantitatively lower (i.e. closer to ‘no problems’). A respondent had caring responsibilities and was not significantly related to any slider values and thus was not included as a covariate in any of the final models.

**Table 4:** OLS regression results for respondent characteristics predicting slider responses to difficulty response options in the mobility domain

Characteristics	Difficulty response option		
	A lot of difficulty	Some difficulty	Slight difficulty
Number of observations	1,161	1,161	1,161
Age (continuous)	− 0.028 (0.024)	0.036 (0.038)	− 0.124 (0.036)***
Female (Ref: male, other, or prefer not to say)	1.357 (0.632)*	3.768 (1.023)***	1.719 (0.955)
English as a second language (Ref: English native speakers)	− 0.500 (0.922)	− 2.286 (1.493)	1.054 (1.394)
With mental health conditions (Ref: no mental health conditions)	− 1.211 (0.890)	− 1.606 (1.441)	− 1.727 (1.345)
With physical health conditions (Ref: no physical health conditions)	− 0.623 (0.750)	− 1.350 (1.214)	− 3.242 (1.134)**
With bachelors or higher degrees (Ref: with education below degree level)	− 0.754 (0.629)	− 0.300 (1.018)	− 1.551 (0.951)
Constant	87.047 (1.155)***	47.174 (1.869)***	32.935 (1.745)***
Adjusted R-squared	0.004	0.011	0.030
*F*-test (Prob > F)	1.80 (.095)	3.06 (.006)	7.01 (.000)

*Note* Standard errors in parentheses

*OLS* ordinary least squares, *Ref* reference category

***p < .001, **p < .01, *p < .05. Each column represents a separate regression model for a single response option

Supplementary analyses excluding any respondent with one or more inconsistency resulted in slight changes to the significance level of some of the individual characteristics in the frequency and severity models, most notably age and education (see Online Resource 4), but overall findings remained consistent.

## Discussion

This study addressed three key questions. Initially, we explored whether respondents’ quantification of different response options reflected their intuitive or linguistic ordering. In general, this was the case, suggesting that the assumed qualitative ordering of these common response options (when presented together) has underlying validity. Nevertheless, there are further takeaways. First, in previous studies, respondents tended to spread out all response options on a numerical scale when evaluating them simultaneously [25, 30]. Therefore, the same response option might appear to have a different numerical value when a number of other options vary. One strength of this study is that respondents were allocated to different arms through randomisation. As a result, we can draw inferences that the labels given to response options influenced numerical interpretation beyond the positioning or order of the options shown. Despite being in the same position within the choice set, ‘occasionally’ had a higher value for respondents than ‘only occasionally’. Similarly, the values across frequency and severity response options differed, with values for ‘often’ being above those for ‘quite a bit’ despite the fact that they were both ordered in fourth position within their respective response option scales.

Second, there was greater quantitative differentiation between some terms than others (e.g. see Fig. 2). For example, the difference between ‘a little bit’ and ‘somewhat’ was smaller than the difference between ‘somewhat’ and ‘quite a bit’. Further, the distance between ‘sometimes’ and ‘occasionally’ was lower than distances between other neighbouring response options. The similarity of interpretation of these two terms has been found elsewhere. Spector [30], in an exercise with students from the University of South Florida, found that ‘sometimes’ and ‘occasionally’ were given the same overall ranking. Our results suggest that people do not simply apply an interval approach to ranking a finite number of response options on the same scale, which has implications for scale design and analysis. Rather than interval-based scoring systems for PROMs, it may support the use of a scoring system which draws upon other information to provide the relative score for a response option, such as the use of item response theory (IRT) or preference-based scoring [31, 32].

Third, as Fig. 2 shows, there is a clear gap in the middle of the quantitative rating scale for the selection of response options tested in this study. This suggests that none of the options tested in this study was really adequate for a Likert scale that requires a mid-point, and it prompts the need for further testing of response options that may better fulfil this role within PROM design (e.g. ‘half the time’).

The second research question was whether domain affected the quantitative value participants placed on response options. We found some support for this. The loneliness domain, which featured a negatively worded question stem (i.e. more is worse) results in response options having a lower numerical interpretation compared to the two positively worded happiness and activities domains. Similar results have been reported elsewhere, with negatively phrased questions receiving lower values on average than positively phrased items [13]. Nevertheless, as we used only one negatively phrased question, it is unclear whether our findings are due solely to the negative phrasing of the item, or something specific about the content of the domain (i.e. loneliness), relative to the comparators.

Our final research question was whether participants’ individual characteristics influenced the way response options were interpreted. The overall effect of individual characteristics was substantially smaller than the effect of domain context, which was a positive finding. However, some characteristics made a difference, and these may have a cumulative impact when multi-item PROMs are completed. Women and older respondents tended to report significantly higher values for a number of response options, especially towards the top of the scale, and this should be taken into consideration in research involving mixed samples. Of particular interest was the effect of ESL on response option interpretation. The labels ‘only occasionally’ (but not ‘occasionally’), ‘somewhat’, and ‘quite a bit’ were interpreted significantly differently by respondents with ESL, with the variation in the interpretation of ‘quite a bit’ being significantly greater for respondents with ESL than those without. Interestingly, the numeric value of ‘quite a bit’ (without a specific domain context) was found to be higher in a Swedish study (mean = 73.5) [23] compared to our findings. Additionally, ‘quite a bit’ was also found to have a higher numerical value (mean = 75.1) in an international study testing the translation equivalence of SF-36 in different countries [28], with the interpretation of ‘quite a bit’ varying from country to country [24, 33]. These previous studies show that country and translation might have an impact on the interpretation of response options, whereas our study further explored the effect of ESL on understanding response options within the same language.

We acknowledge some limitations of the present study. As an online survey recruited through a commercial panel, the data will be subject to concerns over quality. This risk was mitigated through keeping the survey short, careful design (and piloting) of the survey, and dropping logically inconsistent responses for individual questions plus full cases that had at least three cases of inconsistency. As a consequence of keeping the survey short individuals only interpreted response options for one of the three domains. This meant comparison between questions was based on different individuals—while this had the advantage that values given were not being impacted by potential ordering effects—it is a potential limitation in making comparisons. Furthermore, only three domains were explored, which limits the interpretation and generalisation of the findings. The quantitative interpretation of the response options relied upon a visual analogue scale (VAS) scale (our slider), which has well known biases, such as end aversion [34]; the extent to which such a bias may be interacting here with individual characteristics or question domain is unknown.

This study has shown that while respondents quantitatively interpret common response options in a logical way, this interpretation may differ systematically based on domain being measured and certain individual characteristics, and this should be taken into account in PROM design and analysis. Several recommendations can be made based on our findings. First, in PROM design, it is sensible not to mix negatively and positively phrased domain items within the same measure [35]. This is particularly the case within the same multi-item scale (or set of items that are combined to calculate a score). It may be possible to include consistent sets of positively and negatively worded items within a domain (or subscale) of a PROM, when those domains are not then combined to form a total score. However, if these domains use the same response options, then PROM developers should be aware that the same scale may be interpreted differently across domains as a function of negative or positive wording. Further, if a PROM is intended to be valued for use in cost-effectiveness analysis (i.e. is to be ‘preference-based’ [15]), and there is particular need to avoid positively and negatively phrased items within the same PROM. Prototypical items from different domains are typically used together in health state valuation exercises and mixing positive and negative items may lead to a differential quantitative interpretation by respondents. Researchers following this advice will also need to consider whether they want to include wholly positive or negatively phrased items during PROM design. This is an issue that, in our opinion, can be best addressed in collaborative patient and public involvement and engagement work with the PROM’s target population and/or appropriate cognitive debriefing exercises.

Second, in PROM design and analysis, simple PROM scoring systems that rely on an assumption of interval properties of Likert response options should be avoided wherever possible [31]. Instead, methods such as IRT scoring can be used to adjust for uneven distances between response options. If the PROM is to be valued for use in health resource cost-effectiveness analysis and requires item reduction, then it is possible to use the output from IRT analyses to select items that produce the best spread across the latent scale.

Third, our findings can be used to inform the selection of response options for future PROM development, depending on the target research sample. Most successful PROMs are not designed to be used solely in people with English as a first language and so researchers should consider their choice of response options carefully for interpretability across people with ESL during the design stage [16]. For example, if the sample involves participants with ESL then researchers should consider avoiding using the response options ‘somewhat’ and ‘quite a bit’.

## Electronic supplementary material

Below is the link to the electronic supplementary material.

Supplementary material 1 (DOCX 50kb)

Supplementary material 2 (DOCX 22kb)

Supplementary material 3 (DOCX 29kb)

Supplementary material 4 (DOCX 34kb)

## Footnotes

1 This differed to the original response ‘most or all of the time’ in order to explore the quantitative interpretation of ‘most’.
