# Supplementary material for: The role of response domain and scale label in the quantitative interpretation of patient-reported outcome measure response options
Source: Qual Life Res. 2021 Mar 4;30(7):2097–108. doi: 10.1007/s11136-021-02801-9 (PMC8233274; doi:10.1007/s11136-021-02801-9)
Supplement: Supplementary file 1 — Supplementary material 1 (DOCX 50kb) [file 11136_2021_2801_MOESM1_ESM.docx]

**Online Resource 1 – Study survey**

Study about survey answers

Start of Block: Introduction/consent

Q1 Before we start the survey we need to check you are happy to be involved in this research project, and understand why we are collecting this data. This study is all about the questions we might ask people to find out about their health and wellbeing. We want to find out what people think about the different answers or "response choices" used in questions. 

 You will be asked to complete some questions about yourself and your health. Then you will be asked to answer some questions about how you feel or things that you have been able to do. These questions have some possible answers or response choices such as "often". This study is trying to find out what these answers such as "often" mean to people. You will be asked some questions about what your answers mean to you. You will also be asked about other possible answers that you have not selected and what you think they mean. 

 We think that the whole survey will take about 10 minutes. There is a 30 minute time limit. There is a £1.20 payment for completion of the survey. 

 We will keep your Prolific ID in order to pay you. Once we have authorised payment we will not keep data that includes your Prolific ID. The only data we will keep will be anonymised and we will not be able to identify who you are.  

 If you would like to look over further details about the study, these can be found here [Information sheet v2.1](https://scharr.eu.qualtrics.com/CP/File.php?F=F_2mp8SvOolHSDaHH). This has our contact details if you would like to ask any questions. It also has information about how we will look after your data. If you are happy to proceed, then we would like you to answer the consent questions on the next page.      

 If you have any questions about the research please contact Tessa Peasgood on T.Peasgood@sheffield.ac.uk

End of Block: Introduction/consent

Start of Block: Consent - taking part in the project

| 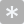 |
| --- |

Q2 Taking part in the project 
 
We are only able to include those people in the study who are happy to provide this consent.

|  | Yes |
| --- | --- |
| I have downloaded, read and understood the project Information Sheet version 2.1, OR I am happy that I understand the project. |  |
| I have been given the opportunity to ask questions about the project by contacting the lead researcher if I want to (full contact details are on the Information Sheet). |  |
| I agree to take part in the project. I understand that this will include completing an online survey that involves tasks about understanding what people mean by words used in answer choices in questionnaires and answering background questions about myself. |  |
| I understand that my taking part is voluntary and that I can drop out from the study before I have received my payment. I understand the researchers may use data collected up until I drop out. I do not have to give any reasons for why I no longer want to take part. |  |
| I understand that for completing this study I will receive a payment of £1.20. I understand that I may not receive my payment if I do not provide a complete response, in line with the standard procedures on Prolific. |  |

End of Block: Consent - taking part in the project

Start of Block: Consent - how my data is used

| 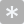 |
| --- |

Q4 How my information will be used during and after the project 


We are only able to include those people in the study who are happy to provide this consent.

|  | Yes |
| --- | --- |
| I understand my Prolific ID will be used only for the purposes of the checking data quality and for the purposes of study payments. They will not be revealed to people outside the project. |  |
| I understand and agree that other authorised researchers will have access to my anonymised data to be used for research purposes and in publications, reports, web pages, and other research outputs. I give permission for the anonymised data that I provide to be shared for the purposes of future research and learning. |  |

End of Block: Consent - how my data is used

Start of Block: Input Prolific ID

| 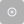 |
| --- |

Q5 Please enter your **Prolific ID** [Note for participants: it can be found at the top of this webpage or when going to your account info]:

________________________________________________________________

End of Block: Input Prolific ID

Start of Block: Demographics 1

Q6 What is your gender?

- Male
- Female
- Other
- Prefer not to say

Q7 What is your age?

________________________________________________________________

| 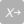 |
| --- |

Q8 Which best describes your ethnic group?

- White British
- Other White
- Asian/Asian British
- Black/African/Carribean/Black British
- Mixed/Multiple ethnic groups
- Other ethnic group
- Prefer not to say

End of Block: Demographics 1

Start of Block: Demographics 2

| 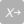 |
| --- |

Q9 What is the highest educational or school qualification you have completed?

- Bachelors or equivalent first degree level qualification or higher
- A-level or equivalent post-secondary level qualification
- GCSE or equivalent secondary school qualification
- None of the above

Q10 In general, would you say your health is:

- Very good
- Good
- Fair
- Bad
- Very bad

Q11 Does your health interfere with your ability to carry out your day-to-day activities?

- Not at all
- A little bit
- Moderately
- Quite a bit
- Extremely

Q12 Do you have a long-standing physical or mental impairment, illness or disability which has been diagnosed by a doctor? (By long-standing we mean anything which has troubled you over a period of at least 12 months or that is likely to trouble you for a period of at least 12 months)

- Yes
- No
- Prefer not to say

Q13 Do you have any of the following conditions?

 Please tick all that apply

- Asthma
- Arthritis
- Heart condition
- Stroke
- Emphysema
- Over-active thyroid
- Under-active thyroid
- Chronic bronchitis
- Liver condition
- Cancer or malignancy
- Diabetes
- Epilepsy
- High blood pressure
- Irritable bowel syndrome or Crohn's disease or Ulcerative Colitis
- Depression
- Generalised Anxiety disorder
- Disability
- Other mental health problem
- Other physical health problem

Q14 Is there anyone, either living with you or not living with you, who is sick, disabled or elderly whom you look after or give special help to?

- Yes
- No

| Page Break |  |
| --- | --- |

Q166 Thank you. 


We are now going to ask you about the health and wellbeing questions that this survey is all about.

End of Block: Demographics 2

Start of Block: RO questions lonely 1 ('only occasionally' participant answer)

Q15 We are going to look at a question about loneliness. 
Please answer this question:          


**Thinking about how things have been over the last 7 days....  I felt lonely**

- none of the time
- only occasionally
- sometimes
- often
- most or all of the time

End of Block: RO questions lonely 1 ('only occasionally' participant answer)

Start of Block: RO questions lonely 1 ('only occasionally' participant answer interpretation)

| 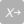 |
| --- |

Q16 We are interested in what your answer to that question "**I felt lonely**" meant to you.   


You gave the answer '**${Q15/ChoiceGroup/SelectedChoices}**'. 


In your opinion, how many times during the last 7 days, does '**${Q15/ChoiceGroup/SelectedChoices}**' refer to?    
Please select the closest response.

- Not even once in the last 7 days
- Once in the last 7 days
- Twice in the last 7 days
- Three times in the last 7 days
- Four times in the last 7 days
- Five times in the last 7 days
- Six times in the last 7 days
- Seven or more times in the last 7 days

End of Block: RO questions lonely 1 ('only occasionally' participant answer interpretation)

Start of Block: Response option questions lonely 2 (random answer only occasionally)

| 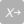 |
| --- |

Q17 We are now going to think about a different possible answer to the question "**I felt lonely**". 


If someone had answered '**only occasionally**' to the question, what do you think this would refer to in terms of number of times during the last 7 days?

- Not even once in the last 7 days
- Once in the last 7 days
- Twice in the last 7 days
- Three times in the last 7 days
- Four times in the last 7 days
- Five times in the last 7 days
- Six times in the last 7 days
- Seven or more times in the last 7 days

End of Block: Response option questions lonely 2 (random answer only occasionally)

Start of Block: Response option questions lonely 2 (random answer often)

| 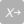 |
| --- |

Q18 We are now going to think about a different possible answer to the question "**I was able to do the things I wanted to do**".      


If someone had answered '**often**', what do you think this answer would refer to in terms of number of times during the last 7 days?

- Not even once in the last 7 days
- Once in the last 7 days
- Twice in the last 7 days
- Three times in the last 7 days
- Four times in the last 7 days
- Five times in the last 7 days
- Six times in the last 7 days
- Seven or more times in the last 7 days

End of Block: Response option questions lonely 2 (random answer often)

Start of Block: Response option questions lonely 2 (random answer sometimes)

| 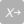 |
| --- |

Q19 We are now going to think about a different possible answer to the question "**I felt lonely**".      


If someone had answered '**sometimes**', what do you think this answer would refer to in terms of number of times during the last 7 days?

- Not even once in the last 7 days
- Once in the last 7 days
- Twice in the last 7 days
- Three times in the last 7 days
- Four times in the last 7 days
- Five times in the last 7 days
- Six times in the last 7 days
- Seven or more times in the last 7 days

End of Block: Response option questions lonely 2 (random answer sometimes)

Start of Block: Response option questions lonely 2 (random answer most of the time)

| 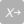 |
| --- |

Q20 We are now going to think about a different possible answer to the question "**I felt lonely**".      


If someone had answered '**most of the time**', what do you think this answer would refer to in terms of number of times during the last 7 days?

- Not even once in the last 7 days
- Once in the last 7 days
- Twice in the last 7 days
- Three times in the last 7 days
- Four times in the last 7 days
- Five times in the last 7 days
- Six times in the last 7 days
- Seven or more times in the last 7 days

End of Block: Response option questions lonely 2 (random answer most of the time)

Start of Block: Intro to slider questions

Q21 The next few questions use sliders to find out how you interpret these different answers or response choices   


We are going to ask you to drag a slider to the position that you think best suits each '**possible answer**' to the question. 


The sliders look like the one below with 0 at one end and 100 at the other (this is just to show you - you don't need to move this one) 


The 0 on the scale represents 'none of the time' and 100 represents 'all of the time'.  

|  | 0 | 10 | 20 | 30 | 40 | 50 | 60 | 70 | 80 | 90 | 100 |
| --- | --- | --- | --- | --- | --- | --- | --- | --- | --- | --- | --- |

|  | 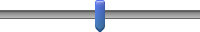 |
| --- | --- |

End of Block: Intro to slider questions

Start of Block: Response option questions lonely 3 (sliders) - only occasionally

| 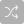 |
| --- |

Q22
**Thinking about how things have been over the last 7 days....  I felt lonely**   
 
Please move the sliders below to show where you think the answer '**sometimes**' falls. 
 
The 0 on the scale represents 'none of the time' and 100 represents 'all of the time'

|  | 0 | 10 | 20 | 30 | 40 | 50 | 60 | 70 | 80 | 90 | 100 |
| --- | --- | --- | --- | --- | --- | --- | --- | --- | --- | --- | --- |

|  | 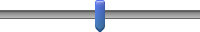 |
| --- | --- |

Q23 **Thinking about how things have been over the last 7 days.... I felt lonely**   


Please move the slide below to show where you think the answer '**only occasionally**' falls. 


The 0 on the scale represents 'none of the time' and 100 represents 'all of the time'

|  | 0 | 10 | 20 | 30 | 40 | 50 | 60 | 70 | 80 | 90 | 100 |
| --- | --- | --- | --- | --- | --- | --- | --- | --- | --- | --- | --- |

|  | 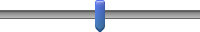 |
| --- | --- |

Q24 **Thinking about how things have been over the last 7 days.... I felt lonely**


Please move the slide below to show where you think the answer '**often**' falls. 


The 0 on the scale represents 'none of the time' and 100 represents 'all of the time'

|  | 0 | 10 | 20 | 30 | 40 | 50 | 60 | 70 | 80 | 90 | 100 |
| --- | --- | --- | --- | --- | --- | --- | --- | --- | --- | --- | --- |

|  | 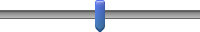 |
| --- | --- |

Q25 **Thinking about how things have been over the last 7 days.... I felt lonely**   


Please move the slide below to show where you think the answer '**most of the time**' falls. 


The 0 on the scale represents 'none of the time' and 100 represents 'all of the time'

|  | 0 | 10 | 20 | 30 | 40 | 50 | 60 | 70 | 80 | 90 | 100 |
| --- | --- | --- | --- | --- | --- | --- | --- | --- | --- | --- | --- |

|  | 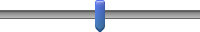 |
| --- | --- |

| Page Break |  |
| --- | --- |

Q26 Thank you.   


We are going to do the same thing again - still using the question:   **Thinking about how things have been over the last 7 days.... I felt lonely**   


But this time we are going to use a different set of answers where we are trying to understand what you think they mean.

| 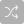 |
| --- |

Q27 **Thinking about how things have been over the last 7 days.... I felt lonely**   


Please use the sliders below to show where you think '**quite a bit**' falls. 


The 0 on the scale represents 'not at all'.

|  | 0 | 10 | 20 | 30 | 40 | 50 | 60 | 70 | 80 | 90 | 100 |
| --- | --- | --- | --- | --- | --- | --- | --- | --- | --- | --- | --- |

|  | 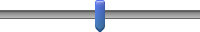 |
| --- | --- |

| 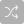 |
| --- |

Q28 **Thinking about how things have been over the last 7 days.... I felt lonely**


Please use the sliders below to show where you think '**very much**' falls. 


The 0 on the scale represents 'not at all'.

|  | 0 | 10 | 20 | 30 | 40 | 50 | 60 | 70 | 80 | 90 | 100 |
| --- | --- | --- | --- | --- | --- | --- | --- | --- | --- | --- | --- |

|  | 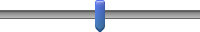 |
| --- | --- |

| 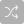 |
| --- |

Q29 **Thinking about how things have been over the last 7 days.... I felt lonely**   


Please use the sliders below to show where you think '**a little bit**' falls. 


The 0 on the scale represents 'not at all'.

|  | 0 | 10 | 20 | 30 | 40 | 50 | 60 | 70 | 80 | 90 | 100 |
| --- | --- | --- | --- | --- | --- | --- | --- | --- | --- | --- | --- |

|  | 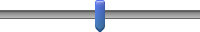 |
| --- | --- |

| 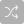 |
| --- |

Q30 **Thinking about how things have been over the last 7 days.... I felt lonely**


Please use the sliders below to show where you think '**somewhat**' falls. 


The 0 on the scale represents 'not at all'.

|  | 0 | 10 | 20 | 30 | 40 | 50 | 60 | 70 | 80 | 90 | 100 |
| --- | --- | --- | --- | --- | --- | --- | --- | --- | --- | --- | --- |

|  | 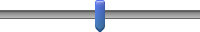 |
| --- | --- |

| 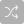 |
| --- |

Q31 **Thinking about how things have been over the last 7 days.... I felt lonely**


Please use the sliders below to show where you think '**some'** falls. 


The 0 on the scale represents 'not at all'.

|  | 0 | 10 | 20 | 30 | 40 | 50 | 60 | 70 | 80 | 90 | 100 |
| --- | --- | --- | --- | --- | --- | --- | --- | --- | --- | --- | --- |

|  | 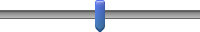 |
| --- | --- |

End of Block: Response option questions lonely 3 (sliders) - only occasionally

Start of Block: RO questions able to do things 1 ('only occasionally' participant answer)

Q32 We are going to look at a question about what you have been able to do. 


We would like you to answer this question:   


**Thinking about how things have been over the last 7 days.... I was able to do the things I wanted to do**

- none of the time
- only occasionally
- sometimes
- often
- most or all of the time

End of Block: RO questions able to do things 1 ('only occasionally' participant answer)

Start of Block: ROQ able to do things 1 ('only occasionally' participant answer interpretation)

| 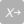 |
| --- |

Q33 We are interested in what your answer to that question meant to you.   


You gave the answer '**${Q32/ChoiceGroup/SelectedChoices}**'    to the question "**I was able to do the things I wanted to do**" 


In your opinion, how many times during the last 7 days, does this  '**${Q32/ChoiceGroup/SelectedChoices}**' refer to?

- Not even once in the last 7 days
- Once in the last 7 days
- Twice in the last 7 days
- Three times in the last 7 days
- Four times in the last 7 days
- Five times in the last 7 days
- Six times in the last 7 days
- Seven or more times in the last 7 days

End of Block: ROQ able to do things 1 ('only occasionally' participant answer interpretation)

Start of Block: ROQ able to do things 2 (random answer only occasionally)

| 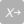 |
| --- |

Q34 We are now going to think about a different possible answer to the question "**I was able to do the things I wanted to do**".   


If someone had answered '**only occasionally**', what do you think this answer would refer to in terms of number of times during the last 7 days?

- Not even once in the last 7 days
- Once in the last 7 days
- Twice in the last 7 days
- Three times in the last 7 days
- Four times in the last 7 days
- Five times in the last 7 days
- Six times in the last 7 days
- Seven or more times in the last 7 days

End of Block: ROQ able to do things 2 (random answer only occasionally)

Start of Block: RO questions able to do things 2 (random answer sometimes)

| 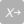 |
| --- |

Q35 We are now going to think about a different possible answer to the question "**I was able to do the things I wanted to do**".   


If someone had answered '**sometimes**', what do you think this answer would refer to in terms of number of times during the last 7 days?

- Not even once in the last 7 days
- Once in the last 7 days
- Twice in the last 7 days
- Three times in the last 7 days
- Four times in the last 7 days
- Five times in the last 7 days
- Six times in the last 7 days
- Seven or more times in the last 7 days

End of Block: RO questions able to do things 2 (random answer sometimes)

Start of Block: RO questions able to do things 2 (random answer often)

| 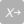 |
| --- |

Q36 We are now going to think about a different possible answer to the question "**I was able to do the things I wanted to do**".   


If someone had answered '**often**', what do you think this answer would refer to in terms of number of times during the last 7 days?

- Not even once in the last 7 days
- Once in the last 7 days
- Twice in the last 7 days
- Three times in the last 7 days
- Four times in the last 7 days
- Five times in the last 7 days
- Six times in the last 7 days
- Seven or more times in the last 7 days

End of Block: RO questions able to do things 2 (random answer often)

Start of Block: RO questions able to do things 2 (random answer most of the time)

| 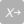 |
| --- |

Q37 We are now going to think about a different possible answer to the question "**I was able to do the things I wanted to do**".   


If someone had answered '**most of the time**', what do you think this answer would refer to in terms of number of times during the last 7 days?

- Not even once in the last 7 days
- Once in the last 7 days
- Twice in the last 7 days
- Three times in the last 7 days
- Four times in the last 7 days
- Five times in the last 7 days
- Six times in the last 7 days
- Seven or more times in the last 7 days

End of Block: RO questions able to do things 2 (random answer most of the time)

Start of Block: RO questions able to do things 3 (sliders) - only occasionally

| 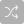 |
| --- |

Q38  **Thinking about how things have been over the last 7 days.... I was able to do the things I wanted to do**


Please use the slider below to show where you think '**only occasionally**' falls. 


The 0 on the scale represents 'none of the time' and 100 represents 'all of the time'.

|  | 0 | 10 | 20 | 30 | 40 | 50 | 60 | 70 | 80 | 90 | 100 |
| --- | --- | --- | --- | --- | --- | --- | --- | --- | --- | --- | --- |

|  | 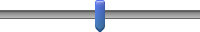 |
| --- | --- |

| 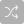 |
| --- |

Q39 **Thinking about how things have been over the last 7 days.... I was able to do the things I wanted to do** 


Please use the slider below to show where you think '**sometimes**' falls. 


The 0 on the scale represents 'none of the time' and 100 represents 'all of the time'.

|  | 0 | 10 | 20 | 30 | 40 | 50 | 60 | 70 | 80 | 90 | 100 |
| --- | --- | --- | --- | --- | --- | --- | --- | --- | --- | --- | --- |

|  | 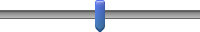 |
| --- | --- |

| 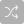 |
| --- |

Q40 **Thinking about how things have been over the last 7 days.... I was able to do the things I wanted to do**


Please use the slider below to show where you think '**often**' falls. 


The 0 on the scale represents 'none of the time' and 100 represents 'all of the time'.

|  | 0 | 10 | 20 | 30 | 40 | 50 | 60 | 70 | 80 | 90 | 100 |
| --- | --- | --- | --- | --- | --- | --- | --- | --- | --- | --- | --- |

|  | 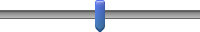 |
| --- | --- |

| 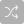 |
| --- |

Q41 **Thinking about how things have been over the last 7 days.... I was able to do the things I wanted to do**


Please use the slider below to show where you think '**most of the time**' falls. 


The 0 on the scale represents 'none of the time' and 100 represents 'all of the time'.

|  | 0 | 10 | 20 | 30 | 40 | 50 | 60 | 70 | 80 | 90 | 100 |
| --- | --- | --- | --- | --- | --- | --- | --- | --- | --- | --- | --- |

|  | 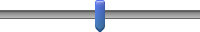 |
| --- | --- |

| Page Break |  |
| --- | --- |

Q42 Thank you.   
We are going to do the same thing again - still using the question   **Thinking about how things have been over the last 7 days.... I was able to do the things I wanted to do**   


But this time we are going to use a different set of answers.  Again we are trying to understand what you think they mean.

| 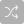 |
| --- |

Q43 **Thinking about how things have been over the last 7 days....  I was able to do the things I wanted to do**       


Please use the slider below to show where you think '**very much**' falls. 


The 0 on the scale represents 'not at all'.

|  | 0 | 10 | 20 | 30 | 40 | 50 | 60 | 70 | 80 | 90 | 100 |
| --- | --- | --- | --- | --- | --- | --- | --- | --- | --- | --- | --- |

|  | 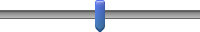 |
| --- | --- |

| 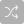 |
| --- |

Q44 **Thinking about how things have been over the last 7 days.... I was able to do the things I wanted to do**   


Please use the slider below to show where you think '**quite a bit**' falls. 


The 0 on the scale represents 'not at all'.

|  | 0 | 10 | 20 | 30 | 40 | 50 | 60 | 70 | 80 | 90 | 100 |
| --- | --- | --- | --- | --- | --- | --- | --- | --- | --- | --- | --- |

|  | 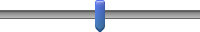 |
| --- | --- |

| 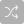 |
| --- |

Q45 **Thinking about how things have been over the last 7 days.... I was able to do the things I wanted to do**

Please use the slider below to show where you think '**a little bit**' falls. 


The 0 on the scale represents 'not at all'.

|  | 0 | 10 | 20 | 30 | 40 | 50 | 60 | 70 | 80 | 90 | 100 |
| --- | --- | --- | --- | --- | --- | --- | --- | --- | --- | --- | --- |

|  | 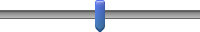 |
| --- | --- |

| 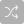 |
| --- |

Q46 **Thinking about how things have been over the last 7 days....  I was able to do the things I wanted to do**      


Please use the slider below to show where you think '**some**' falls. 


The 0 on the scale represents 'not at all'.

|  | 0 | 10 | 20 | 30 | 40 | 50 | 60 | 70 | 80 | 90 | 100 |
| --- | --- | --- | --- | --- | --- | --- | --- | --- | --- | --- | --- |

|  | 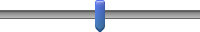 |
| --- | --- |

| 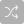 |
| --- |

Q47 **Thinking about how things have been over the last 7 days....   I was able to do the things I wanted to do**      


Please use the slider below to show where you think '**somewhat**' falls. 


The 0 on the scale represents 'not at all'.

|  | 0 | 10 | 20 | 30 | 40 | 50 | 60 | 70 | 80 | 90 | 100 |
| --- | --- | --- | --- | --- | --- | --- | --- | --- | --- | --- | --- |

|  | 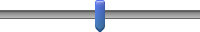 |
| --- | --- |

End of Block: RO questions able to do things 3 (sliders) - only occasionally

Start of Block: RO questions happy 1 participant answer

Q48 We are going to look at a question about happiness. Please answer this question:         


**Thinking about how things have been over the last 7 days....  I felt happy**

- none of the time
- only occasionally
- sometimes
- often
- most or all of the time

End of Block: RO questions happy 1 participant answer

Start of Block: RO questions happy 1 ('only occasionally' participant answer interpretation

| 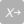 |
| --- |

Q49 We are interested in what your answer to that question "**I felt happy**" meant to you.  

 You gave the answer '**${Q48/ChoiceGroup/SelectedChoices}**'. 


In your opinion, how many times during the last 7 days, does '**${Q48/ChoiceGroup/SelectedChoices}**' refer to?   

 Please select the closest response.

- Not even once in the last 7 days
- Once in the last 7 days
- Twice in the last 7 days
- Three times in the last 7 days
- Four times in the last 7 days
- Five times in the last 7 days
- Six times in the last 7 days
- Seven or more times in the last 7 days

End of Block: RO questions happy 1 ('only occasionally' participant answer interpretation

Start of Block: RO questions happy 2 (random answer often)

| 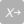 |
| --- |

Q50 We are now going to think about a different possible answer to the question "**I felt happy**". 


If someone had answered '**often**', what do you think this answer would refer to in terms of number of times during the last 7 days?

- Not even once in the last 7 days
- Once in the last 7 days
- Twice in the last 7 days
- Three times in the last 7 days
- Four times in the last 7 days
- Five times in the last 7 days
- Six times in the last 7 days
- Seven or more times in the last 7 days

End of Block: RO questions happy 2 (random answer often)

Start of Block: RO questions happy 2 (random answer only occasionally)

| 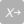 |
| --- |

Q51 We are now going to think about a different possible answer to the question "**I felt happy**" 


If someone had answered '**only occasionally**', what do you think this answer would refer to in terms of number of times during the last 7 days?

- Not even once in the last 7 days
- Once in the last 7 days
- Twice in the last 7 days
- Three times in the last 7 days
- Four times in the last 7 days
- Five times in the last 7 days
- Six times in the last 7 days
- Seven or more times in the last 7 days

End of Block: RO questions happy 2 (random answer only occasionally)

Start of Block: RO questions happy 2 (random answer most of the time)

| 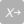 |
| --- |

Q52 We are now going to think about a different possible answer to the question "**I felt happy**" 


If someone had answered '**most of the time**', what do you think this answer would refer to in terms of number of times during the last 7 days?

- Not even once in the last 7 days
- Once in the last 7 days
- Twice in the last 7 days
- Three times in the last 7 days
- Four times in the last 7 days
- Five times in the last 7 days
- Six times in the last 7 days
- Seven or more times in the last 7 days

End of Block: RO questions happy 2 (random answer most of the time)

Start of Block: RO questions happy 2 (random answer sometimes)

| 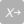 |
| --- |

Q53 We are now going to think about a different possible answer to the question "**I felt happy**"


 If someone had answered '**sometimes**', what do you think this answer would refer to in terms of number of times during the last 7 days?

- Not even once in the last 7 days
- Once in the last 7 days
- Twice in the last 7 days
- Three times in the last 7 days
- Four times in the last 7 days
- Five times in the last 7 days
- Six times in the last 7 days
- Seven or more times in the last 7 days

End of Block: RO questions happy 2 (random answer sometimes)

Start of Block: RO questions happy 3 sliders

| 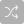 |
| --- |

Q55
**Thinking about how things have been over the last 7 days....  I felt happy**       


Please move the slider below to show where you think the answer '**sometimes**' falls. 


The 0 on the scale represents 'none of the time', and 100 represents 'all of the time'

|  | 0 | 10 | 20 | 30 | 40 | 50 | 60 | 70 | 80 | 90 | 100 |
| --- | --- | --- | --- | --- | --- | --- | --- | --- | --- | --- | --- |

|  | 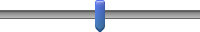 |
| --- | --- |

Q56 **Thinking about how things have been over the last 7 days.... I felt  happy**    


Please move the slider below to show where you think the answer '**only occasionally**' falls. 


The 0 on the scale represents 'none of the time', and 100 represents 'all of the time'

|  | 0 | 10 | 20 | 30 | 40 | 50 | 60 | 70 | 80 | 90 | 100 |
| --- | --- | --- | --- | --- | --- | --- | --- | --- | --- | --- | --- |

|  | 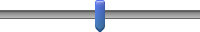 |
| --- | --- |

Q57 **Thinking about how things have been over the last 7 days.... I felt happy**      


Please move the slider below to show where you think the answer '**often**' falls. 


The 0 on the scale represents 'none of the time', and 100 represents 'all of the time'

|  | 0 | 10 | 20 | 30 | 40 | 50 | 60 | 70 | 80 | 90 | 100 |
| --- | --- | --- | --- | --- | --- | --- | --- | --- | --- | --- | --- |

|  | 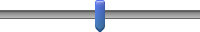 |
| --- | --- |

Q58 **Thinking about how things have been over the last 7 days.... I felt happy**      


Please move the slider below to show where you think the answer '**most of the time**' falls. 


The 0 on the scale represents 'none of the time', and 100 represents 'all of the time'

|  | 0 | 10 | 20 | 30 | 40 | 50 | 60 | 70 | 80 | 90 | 100 |
| --- | --- | --- | --- | --- | --- | --- | --- | --- | --- | --- | --- |

|  | 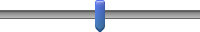 |
| --- | --- |

| Page Break |  |
| --- | --- |

Q59 Thank you.   


We are going to do the same thing again - still using the question:   **Thinking about how things have been over the last 7 days.... I felt happy**    


But this time we are going to use a different set of answers where we are trying to understand what you think they mean.

| 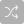 |
| --- |

Q60 **Thinking about how things have been over the last 7 days.... I felt happy**       


Please use the sliders below to show where you think '**quite a bit**' falls. 


The 0 on the scale represents 'not at all'.

|  | 0 | 10 | 20 | 30 | 40 | 50 | 60 | 70 | 80 | 90 | 100 |
| --- | --- | --- | --- | --- | --- | --- | --- | --- | --- | --- | --- |

|  | 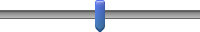 |
| --- | --- |

| 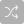 |
| --- |

Q61 **Thinking about how things have been over the last 7 days.... I felt happy**      


Please use the sliders below to show where you think '**very much**' falls. 


The 0 on the scale represents 'not at all'.

|  | 0 | 10 | 20 | 30 | 40 | 50 | 60 | 70 | 80 | 90 | 100 |
| --- | --- | --- | --- | --- | --- | --- | --- | --- | --- | --- | --- |

|  | 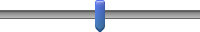 |
| --- | --- |

| 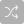 |
| --- |

Q62 **Thinking about how things have been over the last 7 days....I felt happy**       


Please use the sliders below to show where you think '**a little bit**' falls. 


The 0 on the scale represents 'not at all'.

|  | 0 | 10 | 20 | 30 | 40 | 50 | 60 | 70 | 80 | 90 | 100 |
| --- | --- | --- | --- | --- | --- | --- | --- | --- | --- | --- | --- |

|  | 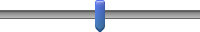 |
| --- | --- |

| 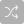 |
| --- |

Q63 **Thinking about how things have been over the last 7 days....I felt happy**       


Please use the sliders below to show where you think '**somewhat**' falls. 


The 0 on the scale represents 'not at all'.

|  | 0 | 10 | 20 | 30 | 40 | 50 | 60 | 70 | 80 | 90 | 100 |
| --- | --- | --- | --- | --- | --- | --- | --- | --- | --- | --- | --- |

|  | 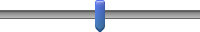 |
| --- | --- |

| 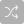 |
| --- |

Q64 **Thinking about how things have been over the last 7 days....I felt happy**       


Please use the sliders below to show where you think '**some**' falls. 


The 0 on the scale represents 'not at all'.

|  | 0 | 10 | 20 | 30 | 40 | 50 | 60 | 70 | 80 | 90 | 100 |
| --- | --- | --- | --- | --- | --- | --- | --- | --- | --- | --- | --- |

| 1 | 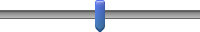 |
| --- | --- |

End of Block: RO questions happy 3 sliders

Start of Block: RO question lonely 1 ('occasionally' participant answer)

Q65 We are going to look at a question about loneliness. 


Please answer this question:          


**Thinking about how things have been over the last 7 days....  I felt lonely**

- none of the time
- occasionally
- sometimes
- often
- most or all of the time

End of Block: RO question lonely 1 ('occasionally' participant answer)

Start of Block: RO question lonely 1 ('occasionally' participant answer interpretation

| 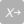 |
| --- |

Q66 We are interested in what your answer to that question "**I felt lonely**" meant to you.   


You gave the answer '**${Q65/ChoiceGroup/SelectedChoices}**'. 


 In your opinion, how many times during the last 7 days, does '**${Q65/ChoiceGroup/SelectedChoices}**' refer to?    
 Please select the closest response.

- Not even once in the last 7 days
- Once in the last 7 days
- Twice in the last 7 days
- Three times in the last 7 days
- Four times in the last 7 days
- Five times in the last 7 days
- Six times in the last 7 days
- Seven or more times in the last 7 days

End of Block: RO question lonely 1 ('occasionally' participant answer interpretation

Start of Block: RO question lonely 2 (random answer occasionally)

| 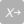 |
| --- |

Q67 We are now going to think about a different possible answer to the question "**I felt lonely**". 

If someone had answered '**occasionally**' to the question, what do you think this would refer to in terms of number of times during the last 7 days:

- Not even once in the last 7 days
- Once in the last 7 days
- Twice in the last 7 days
- Three times in the last 7 days
- Four times in the last 7 days
- Five times in the last 7 days
- Six times in the last 7 days
- Seven or more times in the last 7 days

End of Block: RO question lonely 2 (random answer occasionally)

Start of Block: RO questions lonely 3 (sliders occasionally)

| 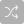 |
| --- |

Q68 We are still thinking about this question:   


**Thinking about how things have been over the last 7 days....  I felt lonely**   


Please move the sliders below to show where you think the answer '**sometimes**' falls. 


The 0 on the scale represents 'none of the time', and 100 represents 'all of the time'

|  | 0 | 10 | 20 | 30 | 40 | 50 | 60 | 70 | 80 | 90 | 100 |
| --- | --- | --- | --- | --- | --- | --- | --- | --- | --- | --- | --- |

|  | 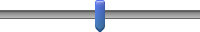 |
| --- | --- |

Q69 **Thinking about how things have been over the last 7 days.... I felt lonely**   


Please move the slide below to show where you think the answer '**occasionally**' falls. 


The 0 on the scale represents 'none of the time', and 100 represents 'all of the time'

|  | 0 | 10 | 20 | 30 | 40 | 50 | 60 | 70 | 80 | 90 | 100 |
| --- | --- | --- | --- | --- | --- | --- | --- | --- | --- | --- | --- |

|  | 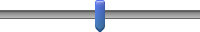 |
| --- | --- |

Q70 **Thinking about how things have been over the last 7 days.... I felt lonely**   


Please move the slide below to show where you think the answer '**often**' falls. 


The 0 on the scale represents 'none of the time', and 100 represents 'all of the time'

|  | 0 | 10 | 20 | 30 | 40 | 50 | 60 | 70 | 80 | 90 | 100 |
| --- | --- | --- | --- | --- | --- | --- | --- | --- | --- | --- | --- |

|  | 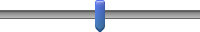 |
| --- | --- |

Q71 **Thinking about how things have been over the last 7 days.... I felt lonely**   


Please move the slide below to show where you think the answer '**most**' falls. 


The 0 on the scale represents 'none of the time', and 100 represents 'all of the time'

|  | 0 | 10 | 20 | 30 | 40 | 50 | 60 | 70 | 80 | 90 | 100 |
| --- | --- | --- | --- | --- | --- | --- | --- | --- | --- | --- | --- |

|  | 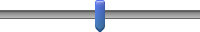 |
| --- | --- |

| Page Break |  |
| --- | --- |

Q72 Thank you.   


We are going to do the same thing again - still using the question:   **Thinking about how things have been over the last 7 days.... I felt lonely**   


But this time we are going to use a different set of answers where we are trying to understand what you think they mean.

| 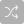 |
| --- |

Q73 **Thinking about how things have been over the last 7 days.... I felt lonely**   


Please use the sliders below to show where you think '**quite a bit**' falls. 


The 0 on the scale represents 'not at all'.

|  | 0 | 10 | 20 | 30 | 40 | 50 | 60 | 70 | 80 | 90 | 100 |
| --- | --- | --- | --- | --- | --- | --- | --- | --- | --- | --- | --- |

|  | 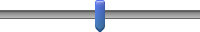 |
| --- | --- |

| 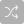 |
| --- |

Q74 **Thinking about how things have been over the last 7 days.... I felt lonely**   


Please use the sliders below to show where you think '**very much**' falls. 


The 0 on the scale represents 'not at all'.

|  | 0 | 10 | 20 | 30 | 40 | 50 | 60 | 70 | 80 | 90 | 100 |
| --- | --- | --- | --- | --- | --- | --- | --- | --- | --- | --- | --- |

|  | 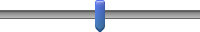 |
| --- | --- |

| 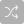 |
| --- |

Q75 **Thinking about how things have been over the last 7 days.... I felt lonely**   


Please use the sliders below to show where you think '**a little bit**' falls. 


The 0 on the scale represents 'not at all'.

|  | 0 | 10 | 20 | 30 | 40 | 50 | 60 | 70 | 80 | 90 | 100 |
| --- | --- | --- | --- | --- | --- | --- | --- | --- | --- | --- | --- |

|  | 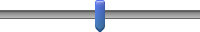 |
| --- | --- |

| 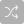 |
| --- |

Q76 **Thinking about how things have been over the last 7 days.... I felt lonely**   


Please use the sliders below to show where you think '**somewhat**' falls. 


The 0 on the scale represents 'not at all'.

|  | 0 | 10 | 20 | 30 | 40 | 50 | 60 | 70 | 80 | 90 | 100 |
| --- | --- | --- | --- | --- | --- | --- | --- | --- | --- | --- | --- |

|  | 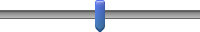 |
| --- | --- |

| 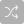 |
| --- |

Q77 **Thinking about how things have been over the last 7 days.... I felt lonely**   


Please use the sliders below to show where you think '**some**' falls. 


The 0 on the scale represents 'not at all'.

|  | 0 | 10 | 20 | 30 | 40 | 50 | 60 | 70 | 80 | 90 | 100 |
| --- | --- | --- | --- | --- | --- | --- | --- | --- | --- | --- | --- |

|  | 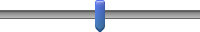 |
| --- | --- |

End of Block: RO questions lonely 3 (sliders occasionally)

Start of Block: RO questions able to do things 1 (occasionally participant answer)

Q78 We are going to look at a question about what you have been able to do. 


We would like you to answer this question:   


**Thinking about how things have been over the last 7 days.... I was able to do the things I wanted to do**

- none of the time
- occasionally
- sometimes
- often
- most or all of the time

End of Block: RO questions able to do things 1 (occasionally participant answer)

Start of Block: ROQ able to do things 1 ('occasionally' participant answer interpretation)

| 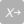 |
| --- |

Q79 We are interested in what your answer to that question meant to you.   


 You gave the answer '**${Q78/ChoiceGroup/SelectedChoices}**'     


In your opinion, how many times during the last 7 days, does '**${Q78/ChoiceGroup/SelectedChoices}**' refer to?

- Not even once in the last 7 days
- Once in the last 7 days
- Twice in the last 7 days
- Three times in the last 7 days
- Four times in the last 7 days
- Five times in the last 7 days
- Six times in the last 7 days
- Seven or more times in the last 7 days

End of Block: ROQ able to do things 1 ('occasionally' participant answer interpretation)

Start of Block: ROQ able to do things 2 (random answer occasionally)

| 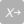 |
| --- |

Q80 We are now going to think about a different possible answer to the question "**I was able to do the things I wanted to do**".   


If someone had answered '**occasionally**', what do you think this answer would refer to in terms of number of times during the last 7 days?

- Not even once in the last 7 days
- Once in the last 7 days
- Twice in the last 7 days
- Three times in the last 7 days
- Four times in the last 7 days
- Five times in the last 7 days
- Six times in the last 7 days
- Seven or more times in the last 7 days

End of Block: ROQ able to do things 2 (random answer occasionally)

Start of Block: RO questions able to do things 3 (sliders occasionally)

| 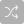 |
| --- |

Q81 **Thinking about how things have been over the last 7 days.... I was able to do the things I wanted to do**      


Please use the slider below to show where you think '**occasionally**' falls. 


The 0 on the scale represents 'none of the time' and 100 represents 'all of the time'.

|  | 0 | 10 | 20 | 30 | 40 | 50 | 60 | 70 | 80 | 90 | 100 |
| --- | --- | --- | --- | --- | --- | --- | --- | --- | --- | --- | --- |

|  | 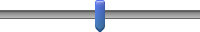 |
| --- | --- |

| 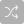 |
| --- |

Q82 **Thinking about how things have been over the last 7 days.... I was able to do the things I wanted to do**   


Please use the slider below to show where you think '**sometimes**' falls. 


The 0 on the scale represents 'none of the time' and 100 represents 'all of the time'.

|  | 0 | 10 | 20 | 30 | 40 | 50 | 60 | 70 | 80 | 90 | 100 |
| --- | --- | --- | --- | --- | --- | --- | --- | --- | --- | --- | --- |

|  | 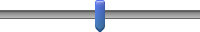 |
| --- | --- |

| 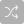 |
| --- |

Q83 **Thinking about how things have been over the last 7 days.... I was able to do the things I wanted to do**   


Please use the slider below to show where you think '**often**' falls. 


The 0 on the scale represents 'none of the time' and 100 represents 'all of the time'.

|  | 0 | 10 | 20 | 30 | 40 | 50 | 60 | 70 | 80 | 90 | 100 |
| --- | --- | --- | --- | --- | --- | --- | --- | --- | --- | --- | --- |

|  | 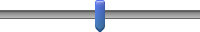 |
| --- | --- |

| 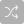 |
| --- |

Q84 **Thinking about how things have been over the last 7 days.... I was able to do the things I wanted to do**
 
Please use the slider below to show where you think '**most of the time**' falls. 


The 0 on the scale represents 'none of the time' and 100 represents 'all of the time'.

|  | 0 | 10 | 20 | 30 | 40 | 50 | 60 | 70 | 80 | 90 | 100 |
| --- | --- | --- | --- | --- | --- | --- | --- | --- | --- | --- | --- |

|  | 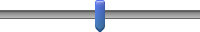 |
| --- | --- |

| Page Break |  |
| --- | --- |

Q85 Thank you.   


We are going to do the same thing again - still using the question   T**hinking about how things have been over the last 7 days.... I was able to do the things I wanted to do**   


But this time we are going to use a different set of answers.  Again we are trying to understand what you think they mean.

| 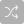 |
| --- |

Q86 **Thinking about how things have been over the last 7 days....  I was able to do the things I wanted to do**       


Please use the slider below to show where you think '**very much**' falls. 


The 0 on the scale represents 'not at all'.

|  | 0 | 10 | 20 | 30 | 40 | 50 | 60 | 70 | 80 | 90 | 100 |
| --- | --- | --- | --- | --- | --- | --- | --- | --- | --- | --- | --- |

|  | 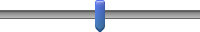 |
| --- | --- |

| 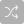 |
| --- |

Q87 **Thinking about how things have been over the last 7 days.... I was able to do the things I wanted to do**   


Please use the slider below to show where you think '**quite a bit**' falls. 


The 0 on the scale represents 'not at all'.

|  | 0 | 10 | 20 | 30 | 40 | 50 | 60 | 70 | 80 | 90 | 100 |
| --- | --- | --- | --- | --- | --- | --- | --- | --- | --- | --- | --- |

|  | 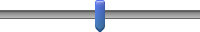 |
| --- | --- |

|  |
| --- |

Q88 **Thinking about how things have been over the last 7 days.... I was able to do the things I wanted to do**

Please use the slider below to show where you think '**a little bit**' falls. 


The 0 on the scale represents 'not at all'.

|  | 0 | 10 | 20 | 30 | 40 | 50 | 60 | 70 | 80 | 90 | 100 |
| --- | --- | --- | --- | --- | --- | --- | --- | --- | --- | --- | --- |

|  |  |
| --- | --- |

|  |
| --- |

Q89 **Thinking about how things have been over the last 7 days....  I was able to do the things I wanted to do**      


Please use the slider below to show where you think '**some**' falls. 


The 0 on the scale represents 'not at all'.

|  | 0 | 10 | 20 | 30 | 40 | 50 | 60 | 70 | 80 | 90 | 100 |
| --- | --- | --- | --- | --- | --- | --- | --- | --- | --- | --- | --- |

|  |  |
| --- | --- |

|  |
| --- |

Q90 **Thinking about how things have been over the last 7 days....   I was able to do the things I wanted to do**      


Please use the slider below to show where you think '**somewhat**' falls. 


The 0 on the scale represents 'not at all'.

|  | 0 | 10 | 20 | 30 | 40 | 50 | 60 | 70 | 80 | 90 | 100 |
| --- | --- | --- | --- | --- | --- | --- | --- | --- | --- | --- | --- |

|  |  |
| --- | --- |

End of Block: RO questions able to do things 3 (sliders occasionally)

Start of Block: RO question happy 1 participant answer (occasionally)

Q91 We are going to look at a question about happiness. 


Please answer this question:         


**Thinking about how things have been over the last 7 days....  I felt happy**

- none of the time
- occasionally
- sometimes
- often
- most or all of the time

End of Block: RO question happy 1 participant answer (occasionally)

Start of Block: ROQ happpy 1 ('occasionally' participant answer interpretation

|  |
| --- |

Q92 We are interested in what your answer to that question "**I felt happy**" meant to you.  

 You gave the answer '**${Q91/ChoiceGroup/SelectedChoices}**'. 


In your opinion, how many times during the last 7 days, does '**${Q91/ChoiceGroup/SelectedChoices}**' refer to?   

 Please select the closest response.

- Not even once in the last 7 days
- Once in the last 7 days
- Twice in the last 7 days
- Three times in the last 7 days
- Four times in the last 7 days
- Five times in the last 7 days
- Six times in the last 7 days
- Seven or more times in the last 7 days

End of Block: ROQ happpy 1 ('occasionally' participant answer interpretation

Start of Block: RO questions happy 2 (random answer occasionally)

|  |
| --- |

Q93 We are now going to think about a different possible answer to the question "**I felt happy**" 


If someone had answered '**occasionally**', what do you think this answer would refer to in terms of number of times during the last 7 days?

- Not even once in the last 7 days
- Once in the last 7 days
- Twice in the last 7 days
- Three times in the last 7 days
- Four times in the last 7 days
- Five times in the last 7 days
- Six times in the last 7 days
- Seven or more times in the last 7 days

End of Block: RO questions happy 2 (random answer occasionally)

Start of Block: RO questions happy 3 sliders (occasionally)

|  |
| --- |

Q94 **Thinking about how things have been over the last 7 days....  I felt happy**     


Please move the slider below to show where you think the answer '**sometimes**' falls. 


The 0 on the scale represents 'none of the time', and 100 represents 'all of the time'

|  | 0 | 10 | 20 | 30 | 40 | 50 | 60 | 70 | 80 | 90 | 100 |
| --- | --- | --- | --- | --- | --- | --- | --- | --- | --- | --- | --- |

|  |  |
| --- | --- |

Q95 **Thinking about how things have been over the last 7 days.... I felt  happy**      


Please move the slider below to show where you think the answer '**occasionally**' falls. 


The 0 on the scale represents 'none of the time', and 100 represents 'all of the time'

|  | 0 | 10 | 20 | 30 | 40 | 50 | 60 | 70 | 80 | 90 | 100 |
| --- | --- | --- | --- | --- | --- | --- | --- | --- | --- | --- | --- |

|  |  |
| --- | --- |

Q96 **Thinking about how things have been over the last 7 days.... I felt happy**      


Please move the slider below to show where you think the answer '**often**' falls. 


The 0 on the scale represents 'none of the time', and 100 represents 'all of the time'

|  | 0 | 10 | 20 | 30 | 40 | 50 | 60 | 70 | 80 | 90 | 100 |
| --- | --- | --- | --- | --- | --- | --- | --- | --- | --- | --- | --- |

|  |  |
| --- | --- |

Q97 **Thinking about how things have been over the last 7 days.... I felt happy**      


Please move the slider below to show where you think the answer '**most of the time**' falls. 


The 0 on the scale represents 'none of the time', and 100 represents 'all of the time'

|  | 0 | 10 | 20 | 30 | 40 | 50 | 60 | 70 | 80 | 90 | 100 |
| --- | --- | --- | --- | --- | --- | --- | --- | --- | --- | --- | --- |

|  |  |
| --- | --- |

| Page Break |  |
| --- | --- |

Q98 Thank you.   


We are going to do the same thing again - still using the question:       **Thinking about how things have been over the last 7 days.... I felt happy**     


But this time we are going to use a different set of answers where we are trying to understand what you think they mean.

|  |
| --- |

Q99 **Thinking about how things have been over the last 7 days.... I felt happy**       


Please use the sliders below to show where you think '**quite a bit**' falls. 


The 0 on the scale represents 'not at all'.

|  | 0 | 10 | 20 | 30 | 40 | 50 | 60 | 70 | 80 | 90 | 100 |
| --- | --- | --- | --- | --- | --- | --- | --- | --- | --- | --- | --- |

|  |  |
| --- | --- |

|  |
| --- |

Q100 **Thinking about how things have been over the last 7 days.... I felt happy**      


Please use the sliders below to show where you think '**very much**' falls. 


The 0 on the scale represents 'not at all'.

|  | 0 | 10 | 20 | 30 | 40 | 50 | 60 | 70 | 80 | 90 | 100 |
| --- | --- | --- | --- | --- | --- | --- | --- | --- | --- | --- | --- |

|  |  |
| --- | --- |

|  |
| --- |

Q101 **Thinking about how things have been over the last 7 days....I felt happy**       


Please use the sliders below to show where you think '**a little bit**' falls. 


The 0 on the scale represents 'not at all'.

|  | 0 | 10 | 20 | 30 | 40 | 50 | 60 | 70 | 80 | 90 | 100 |
| --- | --- | --- | --- | --- | --- | --- | --- | --- | --- | --- | --- |

|  |  |
| --- | --- |

|  |
| --- |

Q102 **Thinking about how things have been over the last 7 days....I felt happy**       


Please use the sliders below to show where you think '**somewhat**' falls. 


The 0 on the scale represents 'not at all'.

|  | 0 | 10 | 20 | 30 | 40 | 50 | 60 | 70 | 80 | 90 | 100 |
| --- | --- | --- | --- | --- | --- | --- | --- | --- | --- | --- | --- |

|  |  |
| --- | --- |

|  |
| --- |

Q103 **Thinking about how things have been over the last 7 days....I felt happy**       


Please use the sliders below to show where you think '**some**' falls. 


The 0 on the scale represents 'not at all'.

|  | 0 | 10 | 20 | 30 | 40 | 50 | 60 | 70 | 80 | 90 | 100 |
| --- | --- | --- | --- | --- | --- | --- | --- | --- | --- | --- | --- |

|  |  |
| --- | --- |

End of Block: RO questions happy 3 sliders (occasionally)

Start of Block: Response option questions get around outside

Q104 Now we are going to look at a different question   
   
**Thinking about how things have been over the last 7 days.... How well were you able to get around outside?**

- no difficulty
- slight difficulty
- some difficulty
- a lot of difficulty
- unable

|  |
| --- |

Q105 We are interested in what people might mean by their answers to the question:
 
**Thinking about how things have been over the last 7 days.... How well were you able to get around outside?**      
 
Please use the sliders below to show where you think '**a lot of difficulty**' falls. 
 
The 0 on the scale represents being 'no difficulty' to get around outside and 100 represents 'unable'

|  | 0 | 10 | 20 | 30 | 40 | 50 | 60 | 70 | 80 | 90 | 100 |
| --- | --- | --- | --- | --- | --- | --- | --- | --- | --- | --- | --- |

|  |  |
| --- | --- |

|  |
| --- |

Q106 **Thinking about how things have been over the last 7 days.... How well were you able to get around outside?**       


Please use the slider below to show where you think '**some difficulty**' falls. 


The 0 on the scale represents being 'no difficulty' to get around outside and 100 represents 'unable'.

|  | 0 | 10 | 20 | 30 | 40 | 50 | 60 | 70 | 80 | 90 | 100 |
| --- | --- | --- | --- | --- | --- | --- | --- | --- | --- | --- | --- |

|  |  |
| --- | --- |

|  |
| --- |

Q107 **Thinking about how things have been over the last 7 days.... How well were you able to get around outside?**       

Please use the slider below to show where you think '**slight difficulty**' falls. 


The 0 on the scale represents being 'no difficulty' to get around outside and 100 represents 'unable'

|  | 0 | 10 | 20 | 30 | 40 | 50 | 60 | 70 | 80 | 90 | 100 |
| --- | --- | --- | --- | --- | --- | --- | --- | --- | --- | --- | --- |

|  |  |
| --- | --- |

End of Block: Response option questions get around outside

Start of Block: Survey ending message

Q165 If you have any comments you would like to make on this survey - please write them below  (optional)

________________________________________________________________

Q108 Thank you for taking the time to complete this survey and helping us with our research. 


Please click 'next' to submit the survey and return to Prolific.

End of Block: Survey ending message
