# Supplementary material for: The role of response domain and scale label in the quantitative interpretation of patient-reported outcome measure response options
Source: Qual Life Res. 2021 Mar 4;30(7):2097–108. doi: 10.1007/s11136-021-02801-9 (PMC8233274; doi:10.1007/s11136-021-02801-9)
Supplement: Supplementary file 2 — Supplementary material 2 (DOCX 22kb) [file 11136_2021_2801_MOESM2_ESM.docx]

**Online Resource 2 – Mean values of slider questions**

| **Lonely (I felt lonely)** | N* | Mean | SD | Min | Max |
| --- | --- | --- | --- | --- | --- |
| Only occasionally | 220 | 16.995 | 9.677 | 0 | 66 |
| Sometimes [only occasionally branch] | 222 | 33.680 | 14.754 | 0 | 92 |
| Often [only occasionally branch] | 220 | 66.800 | 14.512 | 9 | 95 |
| Most of the time [only occasionally branch] | 221 | 86.339 | 10.335 | 19 | 100 |
| Occasionally | 223 | 23.556 | 14.115 | 4 | 78 |
| Sometimes [occasionally branch] | 224 | 32.500 | 15.586 | 0 | 100 |
| Often [occasionally branch] | 223 | 67.709 | 13.826 | 22 | 100 |
| Most of the time [occasionally branch] | 224 | 86.246 | 10.327 | 18 | 100 |
|  |  |  |  |  |  |
| A little bit | 447 | 17.436 | 9.987 | 0 | 70 |
| Somewhat | 447 | 33.143 | 14.977 | 2 | 85 |
| Some | 447 | 30.204 | 12.556 | 0 | 70 |
| Quite a bit | 447 | 58.000 | 17.991 | 0 | 100 |
| Very much | 447 | 83.633 | 10.886 | 13 | 100 |
|  |  |  |  |  |  |
| **Happy (I felt happy)** |  |  |  |  |  |
| Only occasionally | 228 | 24.575 | 12.885 | 3 | 80 |
| Sometimes [only occasionally branch] | 228 | 45.890 | 15.928 | 6 | 100 |
| Often [only occasionally branch] | 226 | 72.566 | 10.222 | 32 | 95 |
| Most of the time [only occasionally branch] | 228 | 86.969 | 10.163 | 22 | 100 |
| Occasionally | 219 | 31.973 | 16.150 | 4 | 80 |
| Sometimes [occasionally branch] | 219 | 46.475 | 16.355 | 8 | 99 |
| Often [occasionally branch] | 219 | 74.046 | 10.208 | 40 | 100 |
| Most of the time [occasionally branch] | 219 | 88.667 | 8.762 | 36 | 100 |
|  |  |  |  |  |  |
| A little bit | 440 | 23.630 | 12.000 | 2 | 81 |
| Somewhat | 440 | 38.630 | 14.777 | 2 | 97 |
| Some | 440 | 36.089 | 13.389 | 0 | 91 |
| Quite a bit | 440 | 62.905 | 16.007 | 1 | 97 |
| Very much | 440 | 83.852 | 15.234 | 0 | 100 |
|  |  |  |  |  |  |
| **Activities (I was able to do the things I wanted)** | |  |  |  |  |
| Only occasionally | 219 | 24.187 | 13.556 | 5 | 90 |
| Sometimes [only occasionally branch] | 219 | 42.685 | 12.756 | 9 | 100 |
| Often [only occasionally branch] | 219 | 72.936 | 10.713 | 24 | 100 |
| Most of the time [only occasionally branch] | 219 | 87.790 | 9.771 | 24 | 100 |
| Occasionally | 219 | 32.667 | 16.874 | 0 | 99 |
| Sometimes [occasionally branch] | 219 | 41.932 | 15.002 | 1 | 100 |
| Often [occasionally branch] | 219 | 73.105 | 11.690 | 20 | 100 |
| Most of the time [occasionally branch] | 219 | 89.795 | 6.213 | 61 | 100 |
|  |  |  |  |  |  |
| A little bit | 452 | 25.058 | 12.176 | 2 | 82 |
| Somewhat | 452 | 39.619 | 16.243 | 0 | 96 |
| Some | 452 | 36.912 | 12.897 | 0 | 96 |
| Quite a bit | 452 | 66.836 | 14.588 | 6 | 100 |
| Very much | 452 | 88.212 | 10.287 | 18 | 100 |
|  |  |  |  |  |  |
| **Mobility (I was able to get around outside with…)** | |  |  |  |  |
| A lot of difficulty | 1162 | 85.712 | 10.572 | 4 | 100 |
| Some difficulty | 1162 | 49.461 | 17.162 | 0 | 92 |
| Slight difficulty | 1162 | 26.732 | 16.187 | 0 | 87 |

*Inconsistent responses were dropped within each group of slider questions where the inconsistency was identified (i.e. frequency slider and severity slider in the happiness, loneliness or activities domain, and difficulty slider in the mobility domain). Missing responses for some questions may lead to within group variations.
