# Supplementary material for: The role of response domain and scale label in the quantitative interpretation of patient-reported outcome measure response options
Source: Qual Life Res. 2021 Mar 4;30(7):2097–108. doi: 10.1007/s11136-021-02801-9 (PMC8233274; doi:10.1007/s11136-021-02801-9)
Supplement: Supplementary file 3 — Supplementary material 3 (DOCX 29kb) [file 11136_2021_2801_MOESM3_ESM.docx]

**Online Resource 3 – Regression results excluding domains as covariates**

**Frequency response options.**

| **Characteristic** | **Only occasionally** | **Occasionally** | **Sometimes** | **Often** | **Most of the time** |
| --- | --- | --- | --- | --- | --- |
| Number of observations✝ | 667 | 660 | 1,330 | 1,325 | 1,329 |
| Age  (continuous) | -0.063  (0.037) | -0.101*  (0.049) | -0.051  (0.033) | 0.051*  (0.026) | -0.0154 (0.0199) |
| Female  (Ref.: Male, other, or prefer not to say) | -0.797  (0.989) | 0.252  (1.285) | 2.430**  (0.895) | 1.737*  (0.688) | 1.862*** (0.528) |
| English as a second language  (Ref.: English native speakers) | 3.252*  (1.454) | 1.500  (1.878) | -0.014  (1.312) | -0.599  (1.008) | -0.844  (0.775) |
| With mental health conditions  (Ref.: no mental health conditions) | -0.625  (1.398) | 0.200  (1.894) | -1.978  (1.292) | 0.145  (0.993) | -0.630  (0.762) |
| With physical health conditions  (Ref.: no physical health conditions) | -0.428  (1.173) | -3.194*  (1.565) | -1.176  (1.078) | -0.642  (0.828) | 0.121  (0.635) |
| With bachelors or higher degrees  (Ref.: with education below degree level) | -2.373*  (0.999) | -3.350**  (1.265) | -0.983  (0.891) | 0.037  (0.685) | -0.00790 (0.526) |
| Constant | 26.11***  (1.897) | 35.86***  (2.360) | 42.60***  (1.679) | 68.34***  (1.289) | 87.48*** (0.990) |
| Adjusted R-squared | 0.015 | 0.024 | 0.008 | 0.004 | 0.006 |
| F-test (Prob>F) | 2.629  (.016) | 3.646  (.001) | 2.786 (.011) | 1.843 (.088) | 2.226  (.038) |

Note. Standard errors in parentheses. ***p<.001, **p<.01, *p<.05. ✝Each column represents a separate regression model for a single response option.

**Severity response options.**

| **Characteristic** | **A little bit** | **Somewhat** | **Some** | **Quite a bit** | **Very much** |
| --- | --- | --- | --- | --- | --- |
| Number of observations✝ | 1,338 | 1,338 | 1,338 | 1,338 | 1,356 |
| Age  (continuous) | -0.126***  (0.0247) | 0.088**  (0.0326) | -0.053  (0.0279) | 0.097**  (0.0343) | 0.047  (0.0261) |
| Female  (Ref.: Male, other or prefer not to say) | 0.006  (0.655) | -1.333  (0.865) | 0.344  (0.741) | 1.506  (0.912) | 2.121**  (0.692) |
| English as a second language  (Ref.: English native speakers) | 0.324  (0.969) | -3.040*  (1.280) | -0.980  (1.097) | -5.992***  (1.350) | -0.406  (1.026) |
| With mental health conditions  (Ref.: no mental health conditions) | 0.378  (0.955) | 1.208  (1.262) | -0.297  (1.081) | 2.579  (1.330) | 0.844  (1.006) |
| With physical health conditions  (Ref.: no physical health conditions) | -0.876  (0.787) | -0.752  (1.039) | -0.139  (0.890) | 0.033  (1.096) | -0.675  (0.830) |
| With bachelors or higher degrees  (Ref.: with education below degree level) | -1.431*  (0.652) | -0.305  (0.861) | 0.616  (0.737) | -1.809*  (0.908) | 0.477  (0.688) |
| Constant | 28.25***  (1.227) | 34.71***  (1.621) | 36.38***  (1.389) | 59.04***  (1.709) | 82.01***  (1.297) |
| Adjusted R-squared | 0.025 | 0.012 | -0.001 | 0.034 | 0.006 |
| F-test (Prob>F) | 6.734 (.000) | 3.678 (.001) | 0.854 (.528) | 8.858 (.000) | 2.269  (.035) |

Note. Standard errors in parentheses. ***p<.001, **p<.01, *p<.05. ✝Each column represents a separate regression model for a single response option.
