# Supplementary material for: The role of response domain and scale label in the quantitative interpretation of patient-reported outcome measure response options
Source: Qual Life Res. 2021 Mar 4;30(7):2097–108. doi: 10.1007/s11136-021-02801-9 (PMC8233274; doi:10.1007/s11136-021-02801-9)
Supplement: Supplementary file 4 — Supplementary material 4 (DOCX 34kb) [file 11136_2021_2801_MOESM4_ESM.docx]

**Online Resource 4 – Sensitivity analyses: regression models with tighter exclusion rules (i.e. respondents with any inconsistencies dropped)**

OLS regression results for respondent characteristics predicting slider responses to frequency and severity response options, with additional exclusions imposed.

|  | **Frequency response options** | | | | | **Severity response options** | | | | |
| --- | --- | --- | --- | --- | --- | --- | --- | --- | --- | --- |
| **Characteristic** | **Only occasionally** | **Occasionally** | **Sometimes** | **Often** | **Most of the time** | **A little bit** | **Somewhat** | **Some** | **Quite a bit** | **Very much** |
| **Number of observations✝** | 548 | 556 | 1,106 | 1,102 | 1,106 | 1,106 | 1,106 | 1,106 | 1,106 | 1,106 |
| **Domain** |  |  |  |  |  |  |  |  |  |  |
| Happiness  (Ref.: loneliness) | 7.131***  (1.134) | 7.572***  (1.458) | 12.060***  (1.036) | 5.926***  (0.825) | 1.658** (0.573) | 5.440***  (0.770) | 5.692***  (1.057) | 5.508***  (0.875) | 4.922***  (1.129) | 2.739***  (0.698) |
| Activities  (Ref.: loneliness) | 7.138***  (1.148) | 7.572***  (1.509) | 8.367***  (1.058) | 5.156***  (0.842) | 2.099*** (0.586) | 6.913***  (0.787) | 6.126***  (1.080) | 6.138***  (0.894) | 9.022***  (1.154) | 4.740***  (0.713) |
| **Respondent characteristics** |  |  |  |  |  |  |  |  |  |  |
| Age  (continuous) | -0.071*  (0.035) | -0.101*  (0.047) | -0.077*  (0.033) | 0.051  (0.026) | 0.007 (0.018) | -0.125***  (0.0246) | 0.085**  (0.0337) | -0.065**  (0.0279) | 0.081**  (0.0360) | 0.042*  (0.0223) |
| Female  (Ref.: Male, other or prefer not to say) | -0.022  (0.951) | 0.242  (1.250) | 2.983***  (0.878) | 2.004**  (0.699) | 1.751*** (0.486) | -0.012  (0.653) | -1.557*  (0.896) | 0.641  (0.741) | 1.493  (0.957) | 1.872***  (0.592) |
| English as a second language  (Ref.: English native speakers) | 3.175*  (1.367) | -0.727  (1.850) | -0.343  (1.283) | -0.003  (1.020) | 0.034 (0.710) | -0.384  (0.954) | -4.403***  (1.310) | -2.174**  (1.084) | -6.220***  (1.399) | -0.087  (0.865) |
| With mental health conditions  (Ref.: no mental health conditions) | 0.334  (1.300) | 0.166  (1.836) | -2.136  (1.242) | -0.292  (0.989) | -0.374 (0.688) | 0.939  (0.923) | 2.181*  (1.267) | 0.206  (1.049) | 2.914**  (1.354) | 1.116  (0.837) |
| With physical health conditions  (Ref.: no physical health conditions) | -0.635  (1.113) | -3.399*  (1.509) | -0.275  (1.044) | -0.148  (0.831) | 0.453 (0.578) | -1.063  (0.776) | -1.114  (1.065) | -0.201  (0.881) | 0.350  (1.138) | -0.447  (0.703) |
| With bachelors or higher degrees  (Ref.: with education below degree level) | -1.706  (0.963) | -1.854  (1.243) | -0.598  (0.879) | 0.210  (0.700) | -0.455 (0.487) | -0.235  (0.654) | 0.694  (0.897) | 1.463**  (0.743) | -1.493  (0.959) | -0.069  (0.593) |
| Constant | 20.13***  (1.889) | 29.65***  (2.429) | 35.70***  (1.726) | 64.25***  (1.372) | 85.73*** (0.956) | 22.96***  (1.283) | 30.16***  (1.762) | 31.98***  (1.458) | 54.79***  (1.882) | 80.81***  (1.163) |
| **Adjusted R-squared** | 0.105 | 0.080 | 0.126 | 0.056 | 0.020 | 0.100 | 0.052 | 0.056 | 0.082 | 0.045 |
| **F-test (Prob>F)** | 9.045 (.000) | 6.988  (.000) | 20.980  (.000) | 9.169  (.000) | 3.828 (.000) | 16.410 (.000) | 8.631  (.000) | 9.182  (.000) | 13.250  (.000) | 7.529  (.000) |

Note. Standard errors in parentheses. ***p<.001, **p<.01, *p<.05. ✝Each column represents a separate regression model for a single response option primarily due to the randomisation in the survey (see Figure 1). OLS: Ordinary Least Squares. Ref.: reference category.

OLS regression results for respondent characteristics predicting slider responses to difficulty response options in the mobility domain, with additional exclusions imposed.

|  | **Difficulty response option** | | |
| --- | --- | --- | --- |
| **Characteristics** | **A lot of difficulty** | **Some difficulty** | **Slight difficulty** |
| **Number of observations✝** | 1,106 | 1,106 | 1,106 |
| Age  (continuous) | -0.037  (0.022) | 0.034  (0.039) | -0.122***  (0.036) |
| Female  (Ref.: Male, other or prefer not to say) | 1.623**  (0.579) | 3.357**  (1.033) | 1.369  (0.965) |
| English as a second language  (Ref.: English native speakers) | 0.106  (0.846) | -2.312  (1.510) | 1.233  (1.410) |
| With mental health conditions  (Ref.: no mental health conditions) | -1.080  (0.818) | -1.082  (1.460) | -1.467  (1.364) |
| With physical health conditions  (Ref.: no physical health conditions) | -0.570  (0.688) | -1.211  (1.228) | -3.094**  (1.147) |
| With bachelors or higher degrees  (Ref.: with education below degree level) | -1.031  (0.578) | -0.318  (1.033) | -1.597  (0.965) |
| Constant | 87.67***  (1.061) | 47.23***  (1.893) | 32.77***  (1.769) |
| **Adjusted R-squared** | 0.011 | 0.008 | 0.028 |
| **F-test (Prob > F)** | 3.019  (.006) | 2.413  (.025) | 6.296  (  .000) |

Note. Standard errors in parentheses. ***p<.001, **p<.01, *p<.05. ✝Each column represents a separate regression model for a single response option. OLS: Ordinary Least Squares. Ref.: reference category.
